# Double-stranded RNA (dsRNA) technology to control forest insect pests and fungal pathogens: challenges and opportunities

**DOI:** 10.1007/s10142-023-01107-y

**Published:** 2023-05-27

**Authors:** Kiran Singewar, Matthias Fladung

**Affiliations:** Thünen Institute of Forest Genetics, 22927 Großhansdorf, Germany

**Keywords:** Forest protection, RNA interference, Forest pest control, Pathogen outbreak, dsRNA pesticide

## Abstract

Climate change alters the seasonal synchronization between plants and respective pests plus pathogens. The geographical infiltration helps to shift their hosts, resulting in novel outbreaks that damage forests and ecology. Traditional management schemes are unable to control such outbreaks, therefore unconventional and competitive governance is needed to manage forest pests and pathogens. RNA interference (RNAi) mediated double-stranded RNA (dsRNA) treatment method can be implemented to protect forest trees. Exogenous dsRNA triggers the RNAi-mediated gene silencing of a vital gene, and suspends protein production, resulting in the death of targeted pathogens and pests. The dsRNA treatment method is successful for many crop insects and fungi, however, studies of dsRNA against forest pests and pathogens are depleting. Pesticides and fungicides based on dsRNA could be used to combat pathogens that caused outbreaks in different parts of the world. Although the dsRNA has proved its potential, the crucial dilemma and risks including species-specific gene selection, and dsRNA delivery methods cannot be overlooked. Here, we summarized the major fungi pathogens and insect pests that have caused outbreaks, their genomic information, and studies on dsRNA fungi-and pesticides. Current challenges and opportunities in dsRNA target decision, delivery using nanoparticles, direct applications, and a new method using mycorrhiza for forest tree protection are discussed. The importance of affordable next-generation sequencing to minimize the impact on non-target species is discussed. We suggest that collaborative research among forest genomics and pathology institutes could develop necessary dsRNA strategies to protect forest tree species.

**Supplementary Information:**

The online version contains supplementary material available at 10.1007/s10142-023-01107-y.

## Introduction

Forests supply a range of ecosystem benefits and products that are necessary to operate the Earth and human community by awning 30% of the world’s land area (Balla et al. 2021). Most importantly, forests are required for harmonizing the climate and hydrologic cycle (Sheil 2018). Forest vegetation is paramount for the atmosphere, supporting wildlife, ecology, and economic gain, yet a fragile and variable part of the ecosystem (Delire et al. 2011). Pathogens like insects and fungi are the most relevant and clear means of threat to forest trees with their accessory influence due to climate change (Linnakoski and Forbes 2019).

Nowadays, noteworthy loss of forest areas has been recorded globally due to pathogen outbreaks that are temperature-dependent, and climate-driven (Bentz et al. 2015). The pathogen eruption urges to development of a possible solution to maintain forest health and global climate change (Roy et al. 2014).

At present, North American and European conifer forests have been facing destructive recurring disease outbreaks (Bentz et al. 2010; Hicke et al. 2016). Such outbreaks disturb the performance of the forest ecosystem, environmental cycle, and economy of a country by losing tourism and timber earnings (Cahyanto et al. 2018; Marini et al. 2017). For example, the warmer climate has helped the intrusive mountain pine beetle (*Dendroctonus ponderosae*) and the southern pine beetle (*D. frontalis*) to enlarge their geographical range in the USA which produces a repeated eruption and is the source of huge tree-dropping (Lesk et al. 2017).

In the last few decades, many traditional measures have been implemented to control pathogen invasions and outbreaks including removal and destroying the infected foliage, cutting of damaged tree parts (Seidl et al. 2016), dispersing forest stands (Roberts et al. 2020), withdrawal of trees, and installation of traps with pheromone and poisoned log (Wermelinger 2004). However, several drawbacks of the management techniques have been raised for the recurring outbreaks (Hlásny et al. 2017).

Plenty of chemical fungicides and pesticides have also been used to knock down the pathogen outbreak over the past years (Okorski et al. 2015). However, these chemical measures are not successful to control the disease or insect attacks over longer periods (Liebhold and Kean 2019). Instead, many of these substances harm the environment, human health, non-target, and beneficial organisms, and favor widespread resistance development (Billings 2011). Therefore, these strategies raised questions on efficacy and finally, are rejected by the public and banned their usage by foresters (Jactel et al. 2019). Hence, the development, as well as the implementation of alternative, novel, and efficient control methods against forest tree pests and pathogens, has been given the highest priority.

In the epoch of plant genomics, RNA interference (RNAi) is promoted as an economic and ecological pest and pathogen magnet strategy (Baum et al. 2007). RNAi is a conserved post-transcriptional gene silencing mechanism present in almost all eukaryote organisms (Szweykowska-Kulińska et al. 2003), triggered by exogenous double-stranded RNA (dsRNA) (Fire et al. 1998). The latest exceptional research articles showed the successful application of dsRNA vaccination for managing forest pests (Rodrigues et al. 2018; Kyre et al. 2019; Dhandapani et al. 2020a), followed by a dsRNA-mediated treatment mechanism.

The “SmartStax® Pro” maize (Mon87411) is the first RNAi-based product from Bayer company, approved by the US government in 2017 and China in 2021 (De Schutter et al. 2022). The dsRNA targeting the *VACUOLAR SORTING PROTEIN* (*SNF7*) gene of the western corn rootworm (*Diabrotica virgifera virgifera*) and the *Bacillus thuringiensis* (Bt) Cry3Bt1 toxin with glyphosate resistance are merged in the maize (Bolognesi et al. 2012). The root damage is controlled by RNAi-dependent silencing of the *SNF7* gene, responsible for the transport of transmembrane proteins, while the Bt-toxin boosts resistance against the target pest (Romeis and Widmer 2020). However, interest in the SmartStax® Pro-maize is decreasing due to its GM-recombinant approach. Furthermore, the first dsRNA product, Ledprona (Rodrigues et al. 2021) applied through spray is currently in the registration process at US Environmental Protection Agency (EPA). It targets proteasome subunit beta type-5 resulting in 90% mortality in the Colorado Potato Beetle *(Leptinotarsa decemlineata*) (Rodrigues et al. 2021). The successful products are promoting scientists and companies to explore the GM-free approach through the exogenous application of dsRNA.

Here, we summarize the records on forest pathogens and/or diseases that have and had caused outbreaks in forest trees including Oak, Conifer, Birch, Poplar, Ash, Beech, Maple, Elm, and Acer. The keywords “forest pathogens” in Google Scholar search were used. Screening different research articles and online records, a total number of 69 insect pests and 80 fungal pathogens could be listed (Table S1 and S2). Although the potential of dsRNA fungi- and pesticides has been shown for many plant pathogens, the very low number of forest insect pests and fungi pathogens for which this method has been examined (Table 1) is surprising. Therefore, this review focuses on aspects of the dsRNA-mediated forest pest and pathogen control and critically evaluates the future potential of RNAi as part of forest management strategies. The collected information can guide forest ecologists and dsRNA enthusiastic to focus on these pathogens. The work can also serve as a valuable source of information for government and private forest owners, and researchers worldwide.

**Table 1:** Overview of RNAi experiments conducted on forest insect pests and fungal pathogens. Respective pests and pathogens including target genes, methods, and final results are summarized. *siRNA*, small interfering RNA; *dsRNA*, double-stranded RNA; *bp*, base pairs

Common name	Scientific name	Target gene	Molecule and size	Method	Aim	Final analysis	Reference
Insect pests							
European gypsy moth	*Lymantria dispar*	*Ocular albinism type 1 (OA1)*	dsRNA & 200—650 bp	Injection	Insecticide	4.80-fold higher mortality	Sun et al. (2019b)
		*Olfactory receptor co-receptor (OrCo)*	dsRNA & 200 bp	Injection	Functional analysis	Low pheromone response of male	Lin et al. (2015)
		*Locus 365, Locus 28,365*	dsRNA & 250 – 208 bp	Feeding	Insecticide	60% reduction in body mass	Ghosh and Gundersen-Rindal (2017)
		*Juvenile hormone epoxide hydrolase (JHEH)*	dsRNA & 513 bp	Oral delivery	Insecticide	Delayed larval development	Wen et al. (2018)
		*Ecdysone receptor* and *ultraspiracle (USP)*	dsRNA & 528 bp	Injection	Functional analysis	Development shortened	Wen et al. (2020)
Emerald ash borer	*Agrilus planipennis*	*COP, IAP*	dsRNA & 247 – 272 bp	Feeding	Insecticide	24 to 78% mortality	Rodrigues et al. (2017c)
		*Heat shock protein (HSP), Shibire (SHI)*	dsRNA & 468 – 483 bp	Feeding	Insecticide	30–90% mortality	Rodrigues et al. (2018)
		*AplaScrB-2*	dsRNA & 475 bp	Injection	Insecticide	Decreased expression of targeted gene	Zhao et al. (2015)
		*SHI, HSP*	dsRNA & 483 – 468 bp	Feeding	Insecticide	69.44% and 46.66% mortality	Leelesh and Rieske (2020)
Asian longhorn beetle	*Anoplophora glabripennis*	28 different genes	dsRNA 314 – 443 bp	Injection	Insecticide	40% and 100% mortality	Dhandapani et al. (2020b)
		*IAP, SNF7, SSK*	dsRNA & 326 – 386 bp	Feeding	Insecticide	75–90% mortality	Dhandapani et al. (2020a)
*Southern pine beetle* *(SPB*)	*Dendroctonus frontalis*	*HSP, SHI, IAP*	dsRNA & 315, 342, 341 bp	Feeding	Insecticide	20–100% mortality	Kyre et al. (2019)
Spruce budworm	*Choristoneura fumiferana*	*Chitin deacetylase (CDA)*	dsRNA & 978 – 706 bp	Injection	Insecticide	Abnormal phenotypes and high mortality	Quan et al. (2013)
		*V-ATPase A*	-	feeding	Lectins mediated dsRNA delivery	48% mortality	Martinez et al. (2021)
Mountain pine beetle	*Dendroctonus ponderosae*	*HSP, SHI, IAP*	dsRNA & 315, 342, 341 bp	Feeding	Insecticide	75–85% mortality	Kyre et al. (2020)
Willow leaf beetle	*Plagiodera versicolora*	*Actin (ACT), Signal recognition particle protein 54 k (SRP54), HSP, SHI, Cactus (CACT),* and *Soluble N-ethylmaleimide-sensitive fusion attachment proteins (SNAP)*	dsRNA expressed in *Escherichia coli*	Bacteria-Mediated	Insecticide	26–82% mortality	Zhang et al. (2019)
Chinese white pine beetle	*Dendroctonus armandi*	*CSP2*	dsRNA	Injection	Insecticide	Reduced development	Li et al. (2018b)
		*DaAqp12L*	dsRNA	Injection	Insecticide	Mortality after cold stress	Fu et al. (2019)
Fungal pathogens							
Chestnut blight	*Cryphonectria parasitica*	*RNA-dependent RNA polymerases (RdRPs)*: *rdr1*, *rdr2*, and *rdr4*,	-	*Agrobacterium*-mediated gene transformation	Functional analysis	No phenotypic differences	Zhang et al. (2014)
Ink Disease	*Phytophthora cinnamomi*	*Glucanase inhibitor protein (GIP)* and *Necrosis-inducing Phytophthora protein 1 (NPP1)*	-	RANi mediated gene knockdown	Functional analysis	Successful gene knockdown	Chahed (2016)
Dutch elm disease	*Ophiostoma ulmi*	*Endopolygalacturonase* (*epg1*)	-	RANi mediated gene knockdown	Functional analysis	Reduction of transcripts	Carneiro et al. (2010)
Brown patch	*Rhizoctonia solani*	*Polygalacturonase (PG)*	-	RANi mediated gene knockdown	Resistance study	Efficient silencing of genes, and suppression of disease	Rao et al. (2019)
Cotton Wilt Disease	*Verticillium dahliae*	*GFP*	siRNA	siRNA transformation through protoplast	Functional analysis	Successful transformation	Rehman et al. (2016)
Pitch Canker	*Fusarium circinatum*	-	-	Spray-induced	-	-	Casero (2021b)
-	*Fusarium solani*	*ZnII)2Cys6-type transcription regulator* and* class V chitin synthase*	-	RANi mediated gene knockdown	Functional analysis	Less virulent	Shanmugam et al. (2017)
-	*Fusarium oxysporum*	*MAP kinase signaling genes (Fmk1, Hog1,* and* Pbs2)*	-	RANi mediated gene knockdown	Functional analysis	Reduces pathogenesis	Pareek and Rajam (2017)
Gray mold	*Botrytis cinerea*	*Lanosterol 14α-demethylase (erg11 or CYP51), Chitin synthase 1 (chs1), Elongation factor 2 (EF2*	dsRNA & 732 bp	Spray Induced gene silencing, petiole adsorption	dsRNA fungicide	Reduce virulence	Nerva et al. (2020)
		*MAP kinase Bmp3*	dsRNA & 427 bp	Drops of dsRNA on leaves	dsRNA fungicide	Delay in conidial germination	Spada et al. (2021)
		*Ergosterol biosynthesis pathway (ERG)*: *erg13, erg11* and *erg1*	dsRNA & 751	Exogenous Application	dsRNA fungicide	Induced systemic protection	Duanis-Assaf et al. (2022)
		*BC1G_04955 (SS1G_02495 homologue), BC1G_04775, BC1G_01592, BC1G_07805 (SS1G_07873 homologue), and BC1G_10306 (SS1G_11912 homologue)*	dsRNA	Foliar application	dsRNA fungicide	Reduced lesion size	McLoughlin et al. (2018)
		*Vacuolar protein sorting 51 (VPS51), dynactin (DCTN1), and suppressor of actin (SAC1)*	dsRNA & 169 bp, 179 bp, and 168 bp	SIGS	dsRNA fungicide	Topical application inhibited plant disease	Qiao et al. (2021)

## RNAi mechanism in insect pests and fungal pathogens

RNAi is one of the gene silencing mechanisms in the cellular post-transcriptional process by preventing mRNA to express and produce a respective protein (Fire et al. 1998). The post-transcriptional process is well known as quelling in fungi and RNAi in animals that have only the aim of controlling the expression of a gene (Dang et al. 2011). In addition, the RNAi pathway in fungi is involved in pathogenesis, and here, the canonical PTGS pathway to control forest fungal pathogens is considered (Torres-Martínez and Ruiz-Vázquez 2017).

**Fig. 1:**
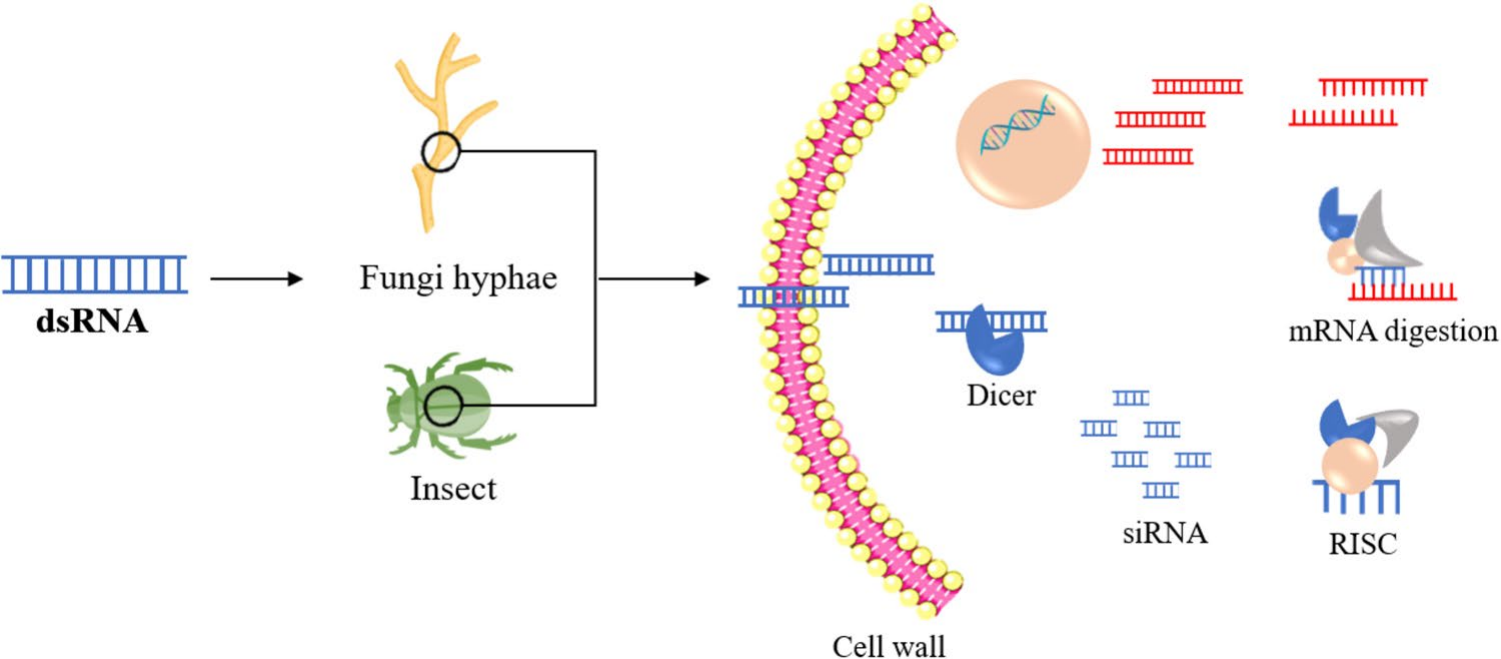
Overview of the general RNAi mechanism in insect pests and fungal pathogens: Exogenous dsRNA triggers the RNAi gene knockdown in insect pests and fungal pathogens. The exogenous dsRNA is recognized and cleaved by the Dicer in siRNA present in the cytoplasm of the insect/fungi cell. Furthermore, the lagging strand of the siRNA degrades and the leading stand gets fastened to the RISC complex. Finally, the attached leading strand of the siRNA to the RISC identifies and degrades the sequence homologs including the mRNA. Eventually, sequence-specific silencing of a gene occurs. Dicer: A ribonuclease; RISC: RNA-induced silencing complex; siRNA: small interfering RNA; Nucleotides of 21 to 24 bases in length

RNA molecules of 18–31 nucleotides (nt) long referred to as small sRNA perform a sequence-specific cleavage or blockage of longer RNAs (Siomi and Siomi 2009) (Fig. 1). Three autonomous sRNA pathways characterized in insects include genome-encoded microRNAs (miRNAs), P-element Induced WImpy testis (PIWI)-interacting (pi)RNAs, and the small interfering RNA (siRNA) (Wang et al. 2006). In contrast, sRNA has been well studied and only miRNA-like RNAs (milRNAs) have been identified in fungi (Ma et al. 2020a). Studies have shown that the piRNA is the undecipherable pathway (Farazi et al. 2008), while miRNA and siRNA pathways are partially and highly sequence-specific respectively to the target RNA (Zhu and Palli 2020).

The RNAi-mediated technology leans on the introduction of dsRNA into the insect and fungi pest to silence an essential target gene, triggering the siRNA pathway (Das and Sherif 2020). Once external dsRNA enters the cell, the Dicer-2 having enzyme ribonuclease III activity processes the dsRNA into siRNAs (21–24 nucleotide duplexes). Furthermore, RNA-induced silencing complex (RISC) including Argonaute2 (AGO2) protein integrates the single antisense strand of siRNAs to form the functioning silencing complex. Finally, the antisense strand of the siRNA guides the RISC complex to select homolog complementary sequences in the mRNA. Finally, the RISC complex carries out the sequence-specific gene silencing by destroying the mRNA (Vogel et al. 2019).

Ago, RdRp, and Dicer are the core components of the RNAi mechanism in fungi, and the loss of one or more of these molecules may affect the functionality of RNAi in fungi (Laurie et al. 2008). In contrast, the sequence homologs of RdRp are almost missing in insects, yet able to trigger the RNAi mechanism when required (Joga et al. 2016).

The conserved eukaryotic pathway, clathrin-mediated endocytosis (CME), controls the uptake of external dsRNA in *Sclerotinia sclerotiorum*, a crop fungal pathogen observed with fluorescence-labeled dsRNA absorbed by the pathogen (Kaksonen and Roux 2018; Wytinck et al. 2020a). Many insects including the model organism *Drosophila* incorporate the CME mechanism (Wytinck et al. 2020b). Systemic RNAi deficient-1 (Sid-1) transmembrane proteins are involved in sRNA uptake in some insects (Cappelle et al. 2016), whereas fungi are completely dependent on CME due to the absence of Sid-1 homologs (Wytinck et al. 2020b).

## dsRNA delivery methods in woody plants

**Fig. 2:**
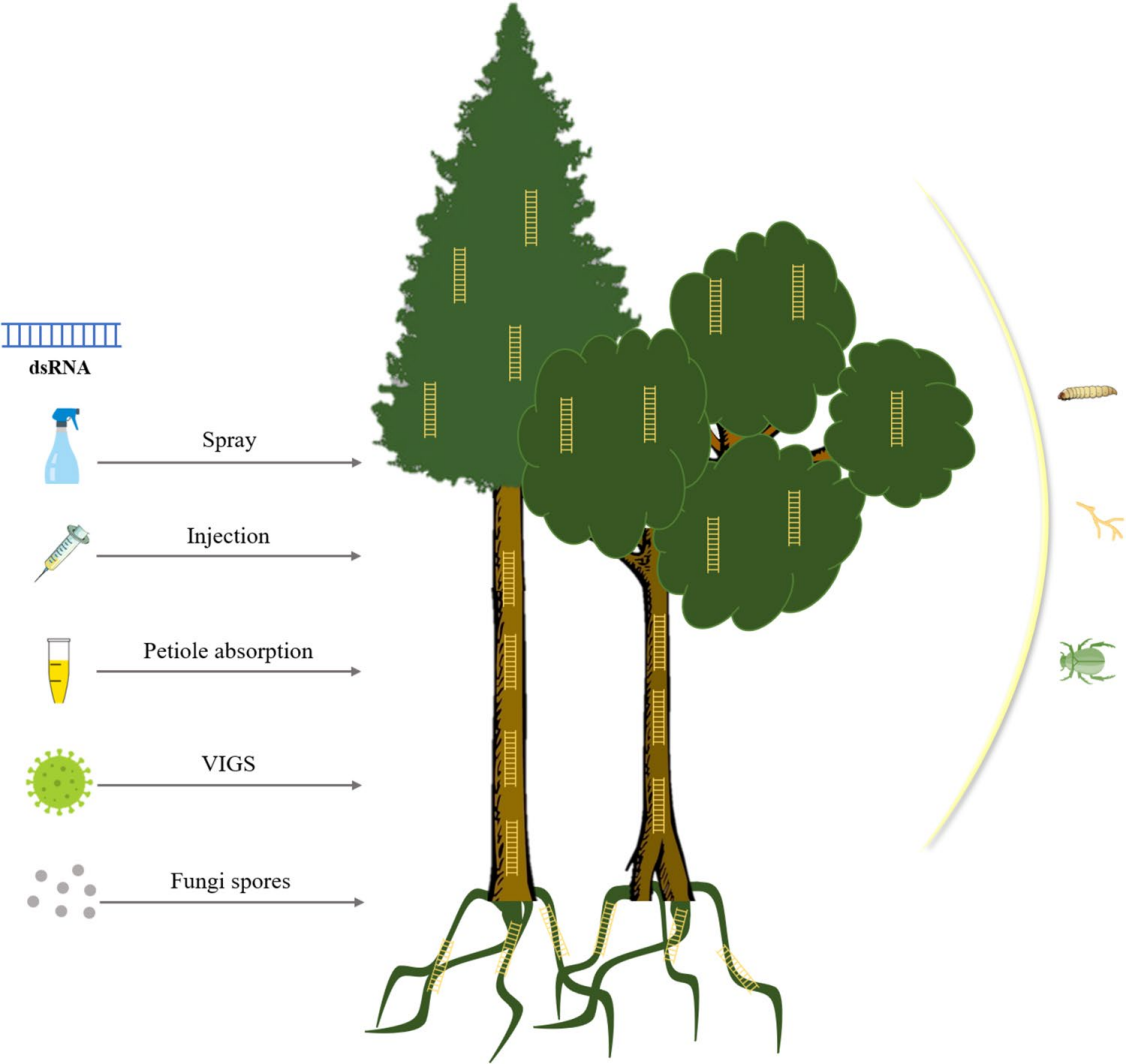
dsRNA mediated woody plant protection from insect pests and fungal pathogens: The left side of the figure shows recombinant (GMO) and non-recombinant (non-GMO) dsRNA delivery approaches to control pests and pathogens. Once the dsRNA reaches tree roots, wood stems, or leaves, most probably, pests and pathogens consume it. Integration of food containing dsRNA triggers the gene silencing mechanism leading to the death of objected pests and pathogens. dsRNA: double-stranded RNA; VIGS: virus-induced gene silencing

Several methods to deliver exogenous dsRNA against insect pests and fungal pathogens have been explored in crop plants and summarized. Generally, spraying, infiltration, injection, spreading, and root soaking methods have been widely used for the exogenous dsRNA of direct applications for plants (Das and Sherif 2020). While, many researchers had also used bacterial, fungal, and viral-mediated dsRNA delivery against various crops. However, studies on exogenous applications of dsRNA on woody plants and to protect against insect pests and fungi are rare. Hence, studies on different exogenous dsRNA delivery methods in woody plants and against various insect pests as well as fungal pathogens are discussed (Fig. 2).

### Spray-induced gene silencing

The latest spray-induced gene silencing technique (SIGS) of RNAi focuses on external applications of dsRNA via sparing on the plant leaves and stems of forest trees (Wang and Jin 2017). Potentially, SIGS is considered an elegant key to controlling forest pests and pathogens as well as concerns genetically modified (GM) organisms and off-target effects of pesticide application (Abdellatef et al. 2021). The targeted fungi and insects consume the sprayed dsRNA and eventually process it using their own RNAi mechanism to initiate the silencing of the target gene. Considering the sequence- and species-specific targeting of the dsRNA on the pathogen and pest followed by the GM-free method makes a unique and agroecosystem-friendly defense approach (Wang and Jin 2017). Some successful studies on the foliar application of dsRNA against forest fungal pathogens from the genus *Botrytis* and *Fusarium* have been tested. A significant reduction in growth and infection was observed in the necrotrophic fungal pathogen *Botrytis cinerea* after applying the dsRNA targeting the *DICER-LIKE1* and *DICER-LIKE2* genes on the surface of fruits, vegetables, and flowers (Wang et al. 2016). Koch and coworkers (Koch et al. 2016) synthesized dsRNA targeting *ERGOSTEROL BIOSYNTHESIS* genes (*CYP3*, *CYP51A*, *CYP51B*, *CYP51C*) of the fungal pathogen *Fusarium graminearum.* Foliar application of the dsRNA was displayed locally as well as distal growth inhibition of the pathogen in the detached leaves. The study further demonstrated that the pathogen consumes the sprayed *CYP3*-dsRNA, transported through the plant vascular system, which is further diced into siRNA by a *DICER-LIKE* gene and controlled the growth of the pathogen in distal tissues (Koch et al. 2016). The RNAi-induced silencing lasted only for 9 h when a dsRNA targeting the *MYOSIN5* gene was sprayed on *F. asiaticum*, the further author suggested studying the SIGS mechanism in more detail (Song et al. 2018). Similarly, a project is in progress to develop a SIGS method to control the grape powdery mildew (*Erysiphe necator*). Three fungal pathogen genes including Dicer-like proteins 1 and 2 followed by *CYP51* are being targeted by spraying complementary dsRNA molecules on the grape leaves (Hahn and Mahlein 2022).

Foliar application of dsRNA or any pesticide, as well as fungicide, is the most traditional way to protect forest tress and crops from serious loss. However, only about 0.4% of the active substances get through the targeted pest and pathogens, suggesting a huge pesticide and fungicide deprivation (Berger and Laurent 2019). Hence, other possible alternatives or simultaneous approaches are mentioned below that can be considered for dsRNA delivery.

### Petiole abortion and trunk injection

The RNase-free habitat of phloem is ideal for dsRNA or siRNA delivery and stability which transports organic compounds, food, and silencing signals in plants (Buhtz et al. 2008). Consistency in dsRNA supply is important for gene silencing, thus dsRNA delivery via irrigation and trunk injection might be efficient compared to spray to control chewing, piercing-sucking, wood-boring, and disease-causing forest pests, and pathogens (Berger and Laurent 2019). Externally applied dsRNA through petiole abortion and trunk injection could spade across the different parts of the plants (Andrade and Hunter 2016).

A 500 base pair long green fluorescent protein (GFP) coding hairpin (hp) RNA was delivered only once through the trunk injection (with a 1-ml syringe without a needle) in domestic apple and common grapevine. A high accumulation of hpRNA was observed on the first day while decreased on the third and tenth days after the northern blot analysis of RNA extracted from leaves. Notable, the hpRNA was injected into the wood base on which the common grapevine plant was grafted. Rapid uptake and systemic transport of the exogenous hpRNA were observed in both woody plants. Moreover, the transportation of hpRNA through the grafting junction was not affected in the common grapevine. Further, an Eppendorf tube containing hpRNA solution was applied through the protruding cut stump of the petiole. The complete hpRNA solution from the tube was efficiently consumed by both plants within a day most probably through capillary forces and vasculature flow (Dalakouras et al. 2018). The petiole absorption and truck injection approach could be of interest to the conifer trees where spraying on life might not work.

Moreover, the dsRNA and siRNAs were anchored in the citrus plants for 57 days and 4 months, respectively when soaked with dsRNA suspension (Hunter et al. 2012). Furthermore, the dsRNA in leafhoppers and psyllids was active just for 5 -8 days after feeding on the citrus plants treated with dsRNA, while elevated mortality in Asian corn borer (*Ostrinia furnacalis*) was detected after the dsRNA targeting *KUNITZ-TYPE TRYPSIN INHIBITORS* (*dsKTI*) irrigated in maize plants (Li et al. 2015). The confirmation of Emerald Ash Borer dsRNA integration through plant tissue was observed using confocal microscopy (Pampolini and Rieske 2020). The dsRNA injected into the trunk through injectors reaches easily into the nuclease-free phloem region of the tree, reaches out quickly to the shoots and roots of the tree, and the signals last for a longer time (Dalakouras et al. 2018). Various companies are manufacturing injectors like Arborjet that help to insert the dsRNA into the trees (https://arborjet.com/research/). Although implementing such technology by injecting each tree does not sound practical, we still believe that innovative techniques could be important to study the effect of synthesized dsRNA. According to the author’s knowledge, most of the dsRNA vaccination studies were conducted only on forest insect pests, however, mentioned techniques could help us to understand the application and viability of the same dsRNA when delivered through trees. Thus, the positive effect of RNAi could be implemented on forest pests and pathogen protection.

Such studies on the exogenous delivery of dsRNA to initiate RNAi could be used to examine the efficiency of dsRNA designed for the suppression of pests and pathogens in woody plants. Petiole absorption and truck injection could be the better alternatives to protect woody plants against fungal pathogens and chewing insect pests.

### Root soaking

In a recent study, Bragg and Rieske (Bragg and Rieske 2022) displayed the consumption, and movement of the exogenous dsRNA when applied through root soaking to the seedlings of *Quercus alba*. The roots of the *Q. alba* seedlings were introduced to necked dsRNA and sampled (stem and leaf) four times (1, 3, 5, and 7 days post-introduction) for RNA extraction. Root uptake of the dsRNA was examined by gel electrophoresis as well as Sanger sequencing to confirm the retrieval of dsRNA soaked by *Q. alba* seedlings and its transport to the stem and leaves. Over time, the accumulation of dsRNA was decreased, however, it was not statistically significant and the stability was constant until 7 days. It is the first study that has used a forest tree species in the experiment and manifested the RNAi-mediated pest management strategy. The proposed method still needs further laboratory as well as greenhouse trials to implement it in natural forests. However, the root soaking method could serve as a valuable method.

### Bacteria- and virus-mediated dsRNA delivery

Elevated efficiency of RNAi was observed in the Asian long-horned beetle (*A. glabripennis*) when the dsRNA was provided through bacterial culture targeting a certain gene (Dhandapani et al. 2020a). The dsRNA aiming *SIGNAL RECOGNITION PARTICLE 54* and *ACTIN* genes were expressed in heat-killed bacterial culture provided to the leaf beetle *Plagiodera versicolora* (Coleoptera: Chrysomelidae) generating significant mortality (Zhang et al. 2019).

A substantial quantity of dsRNA will be needed for forest trees. This can be achieved by *Escherichia coli* strains like HT115(DE3), containing a plasmid construct with the removal of the RNase III gene with a T7 expression vector (Ma et al. 2020b). An engineered bacterium was developed by a biotechnology company; Apse RNA Containers TM (ARCs) could produce a mass amount of dsRNA in the environment which can be used for targeted applications (Joga et al. 2016). Bacteria expressing dsRNA are comparatively much cheaper than the kit-mediated synthesis. However, the release of bacteria into the environment could also pose unintended risks. Although *Escherichia coli* strains, e.g., HT115 (DE3), are save laboratory strains, it is important to reduce potential risks following the release bacteria, e.g., uncontrolled spread. Once released, it is nearly impossible to fetch back the bacteria, and it is possible that unintended risks arise from, e.g., horizontal transfer of the T7 expression vector into other bacterial species. Also, it must be ensured that the released bacteria don’t carry any disease-causing or infectious genes.

The RNAi in the insects could be triggered also with viruses, where the infection-causing genes from the viruses are removed and a gene targeting a specific dsRNA is ligated. The process is called virus-induced gene silencing (VIGS) which uses recombinant virus vectors expressing the desired gene to be delivered to the plants against insect pests and fungal pathogens. Plant viruses use phloem for transport; thus, pest and pathogen management through such a system could be a considerable approach (Kolliopoulou et al. 2020). However, the use of VIGS is limited to laboratory studies. Forest pest and pathogen management through VIGS-mediated dsRNA have not been tested yet, mostly due to rigorous environmental regulation.

Several attempts have been made to control agricultural pathogens; however, so far, only a couple of studies attempted RNAi methods to control forest pathogens. Different dsRNA targeting different genes of the insect pests (mostly Coleoptera family members), as well as fungal pathogens, were synthesized. However, applications and implementation on forest trees are rare. According to our knowledge, all the above-mentioned dsRNA delivery methods have not been tested in forest trees for pests and pathogens ever. Studies on dsRNA delivered through artificial diet (feeding), and injection are common among forest insect pests (Joga et al. 2021). Moreover, none of the forest fungal pathogens have ever been considered, except for some pathogens from the genus *Fusarium* and *Botrytis* which are vital pathogens for crops (Koch et al. 2016; Bennett et al. 2020).

Fungi-induced gene silencing has been discussed in a separate section with a new approach, titled ‘mycorrhiza mediated gene silencing’.

## dsRNA fungi- and pesticide for forest insect pests and fungal pathogens

An artificially provided sequence-specific dsRNA can activate RNAi silencing mechanism as PTGS in insect pests and fungal pathogens (Wynant et al. 2014a). Such artificial dsRNA inoculation was proposed to cause functional loss of the targeted gene since the insect and fungal pathogens initiate their auto-immune siRNA-mediated RNAi defense mechanism upon dsRNA uptake. Here, inoculated dsRNA misleads the antiviral defense mechanism, thus aiming at an endogenous mRNA of the host pest and pathogens (Vogel et al. 2019).

### dsRNA pesticide for forest insect pests

In the last few years, intensive studies have shown the efficiency of dsRNA against wood-boring forest pests like southern pine beetle, mountain pine beetles, emerald ash borer, Asian longhorn beetles, and Chinese white pine beetle (Joga et al. 2021). The feeding of many candidate dsRNAs targeting, *INHIBITOR OF APOPTOSIS* (*IAP*), *HEAT SHOCK 70-kDa PROTEIN COGNATE 3* (*HSP*), *SHIBIRE* (*SHI*), and injecting the *β-FRUCTOFURANOSIDASE-ENCODING-2* (*AplaScrB-2*) genes resulted in more than 80% of mortality to the emerald ash borer (Rodrigues et al. 2017a, b, 2018). A considerable difference in the efficiencies of dsRNA delivery through feeding and injecting has been observed in the Asian long-horned beetle. The dsRNAs of *IAP*, *SNF7*, and *SNAKESKIN* (*SSK*) genes when delivered through feeding, the mortality was 90%, 75%, and 80% respectively, in contrast, 100%, 50%, and 40% of mortality were observed after injecting the dsRNA (Dhandapani et al. 2020a, b). Both studies suggest that dsRNA delivery methods play a vital role in testing the dsRNAs and to develop the vaccination. After feeding the HSP and SHI dsRNA to the Southern pine beetle, 100% and 86.67% mortality had observed (Kyre et al. 2019), while around 85% and 80% mortality was observed in the mountain pine beetle even with comparatively low concentration (Kyre et al. 2020), suggesting the sensitivity also differ among the species of the beetle with the same candidate genes.

The dsRNA-mediated strategy was tested against one of the most forest trees devastating and demanding, the gypsy moth (*Lymantria dispar*), a forest pest belonging to lepidopteran insects. The uncharacterized genes of the gypsy moth were chosen to design the dsRNA using transcriptome sequencing data. Bacteria-expressing and in vitro synthesized dsRNA were provided to the pests through an artificial diet resulting in a reduction in body weight (Ghosh and Gundersen-Rindal 2017). In another study, the removal of the *OCULAR ALBINISM TYPE 1* (*OA1*) by injecting dsRNA into gypsy moth larvae showed an elevated 4.80-fold mortality compared to the control (Sun et al. 2019a). Although the gypsy moth larva did not reach the optimum mortality to use such genes as dsRNA biopesticide, it is clear that the RNAi mechanism is functioning in the pest. Therefore, the selection of the right candidate genes along with addressing the molecular factors that affect the efficiency of dsRNA need to be considered.

Injecting the *CHITIN DEACETYLASE 2* (*CDA2*) dsRNA into larvae or pre-pupae of eastern spruce budworm resulted in high mortality and inability to shed the old cuticle suggesting its important role in the molting process (Quan et al. 2013). Furthermore, a dsRNA binding domain caring a fusion protein consisting of the Galanthus nivalis agglutinin (GNA) was developed and the study showed faster uptake of artificial dsRNA combined with lectin (Martinez et al. 2021).

The eucalyptus gall wasp is one of the devastating insect pests in India affecting several million hectares of eucalyptus trees. Authors had amplified the partial *CHITIN SYNTHASE* gene from the wasp and further planned to design and test the efficiency of dsRNA against it (Nambiar-Veetil et al. 2011). In another study, cost-effective production and bacteria-mediated feeding of dsRNA complementary to *ACTIN*, *SIGNAL RECOGNITION PARTICLE PROTEIN 54 k* (*SRP54*), *CACTUS* (*CACT*), *HSC*, *SHI*, and SOLUBLE *N-ETHYLMALEIMIDE-SENSITIVE FUSION ATTACHMENT PROTEINS* (*SNAP*) genes were attempted to control the willow leaf beetle. Successful RNAi-mediated silencing of all five genes had observed by feeding the dsRNA expressed in bacteria (Zhang et al. 2019).

It is well known that the beetles (*Coleoptera*) are very sensitive to the RNAi-mediated dsRNA and with some more efforts, researchers are not far enough to produce the dsRNA species-specific pesticide. In contrast, the lepidopteran insects are extremely stubborn to RNAi and difficult to produce an effective dsRNA bio-pesticide against them. Cellular uptake, intracellular transportation, and degradation of artificial dsRNA reduce the applicability of RNAi within these insect pests (Martinez et al. 2021). Noticeably, all the described studies were carried out either by feeding or injecting the artificial dsRNA into the insect pest without spraying them on the plant. It would be of interest to observe, whether the efficiency of the dsRNA remains uniform or varies, and such studies could provide an overview of the applicability of dsRNA in actual forest pest management.

### dsRNA fungicide for forest fungal pathogens

A functional study of the *RdRp*, a genetic regulator RNAi in the forest fungal pathogen chestnut blight fungus, *Cryphonectria parasitica* was carried out hypothesizing its role in antiviral defense. However, the knockdown of the *RdRp* could not show any phenotype alteration in the strain (Zhang et al. 2014). Functional study of *GLUCOSE INHIBITOR PROTEIN* (*GIP*), *ARGININE-X-LUCINE-X- ARGININE* (*RXLR*), and *NECROSIS-INDUCING PHYTOPTHORA PROTEIN 1* (*NPP1*) synthesized by *Phytophthora cinnamomi* (Ink disease) during the infection were performed using RNAi technique. The results speculated on the cellular synthesizing destination of both GIP and NPP1 proteins and promoted using dsRNA to control the pathogen (Chahed 2016; Boughanmi 2020). The *Ophiostoma novo-ulmi* causing eradication of the elm tree population throughout North America uses pectinolytic polygalacturonase (epg1) enzymes as a virulence factor. A significant reduction in transcript levels was observed in the transformants using the RNAi technique (Carneiro et al. 2010).

Species of the genus *Rhizoctonia* cause infections in many crop plants like rice making it an economically important pathogen. However, the pathogen also causes significant damage to the forest trees, which is not very well studied. relatively little is known about these fungi and their effects on trees in forestry production. *Rhizoctonia* causing the disease in forest nurseries and tree seedlings has been documented making the fungus an important forest pathogen (Hietala et al. 1996). A *PATHOGENICITY MAP KINASE 1* (PMK1) gene construct for the *Rhizoctonia solani* was transformed into the rice to activate the RNAi-mediated control of the pathogen resulting in a significant reduction of infection (Tiwari et al. 2017). Further, all the key genes required for RNAi activation were characterized to develop the dsRNA-mediated vaccines to control the pathogen (Rao et al. 2019).

The generalist fungal pathogen, *Verticillium dahliae* causes infection in over 200 crop and woody plants (Xiong et al. 2015). Successful delivery of multiple siRNAs through protoplast transformation was carried out in *V. dahliae* to find out the efficiency of siRNA as well as protoplast transformation. Delivered four siRNA were able to successfully silence the transcription *ACTIVATOR OF ADHESION* (*VTA2*) genes in the pathogen (Rehman et al. 2016) promoting the potential of dsRNA in controlling the fungal pathogens.

The pine pitch canker (PPC) caused by the pathogenic fungus *Fusarium circinatum* cannot be controlled in adult trees resulting in a huge loss in forest ecology. A recent project has been initiated aiming to control the pathogen through developing RNA interference (RNAi) strategies (Casero 2021a). More such projects could help to improve our understanding of these forest pathogens and also could develop dsRNA strategies to control them.

The functional role of ZnII)2Cys6-type transcription regulator and *CLASS V CHITIN SYNTHASE* genes were studied through hpRNA-mediated RNAi in *Fusarium oxysporum* and *F. solani*. Phenotypic analysis showed reduced virulence and altered physiological characteristics after the silencing of both genes. Such target genes could be useful to study and develop dsRNA fungicide to manage Fusarium wilt (Shanmugam et al. 2017).

*Botrytis cinerea* spores were able to uptake the fluorescein-labeled dsRNA when incubated together for 12 h. Furthermore, consumed dsRNA targeting the *DICER-LIKE* gene was able to silence the genes as well as reduce the intensity of plant infection when applied to plants (Wang et al. 2016). In another study dsRNA processed with *Fusarium graminearum* conidia showed a knockdown of selected ergosterol biosynthesis genes in the fungi (Bennett et al. 2020). The study shows that *Fusarium* spp., considered important forest pathogens are capable of uptaking the external dsRNA. All the studies have shown that fungi can uptake the environmental dsRNA and carry out RNAi-dependent gene silencing.

All these research articles suggest that many forest fungal pathogens are sensitive to RNAi mechanisms and could be used to develop dsRNA biocontrol. Furthermore, almost all the studies, that have been done on various forest fungal pathogens were either for functional analysis or for crop plants. Therefore, we recommend scientific communities also consider RNAi-mediated studies to develop dsRNA-based fungicides to protect forest trees.

## Challenges in dsRNA development for forest insect pests and fungal pathogens

### Cultivation of forest pests and pathogens

The re-emergence or introduction and spread of any pest and pathogen to a brand-new area, due to climate change or transfer of plant material is a rapidly growing challenge for forest pathologists (Garbelotto and Pautasso 2012; Carvajal-Yepes et al. 2019). Moreover, rearing some pests and pathogens in the laboratory for studies could be very challenging due to food, environmental, and physiological factors (Leppla 2008). Although, around a hundred thousand fungal species have been described, among these tens of thousands of species endure uncultured, or are uncooperative to culture (Rämä and Quandt 2021). Some specialized pests and pathogens need several years of dedication, patience, persistence, observations, and well organization for the successful development of the rearing method (Leppla 2008). Rearing of the pest and pathogens can have unwanted side effects by laboratory adaptation, and artificial diet, therefore providing a diet similar to their environment is vital (Sørensen et al. 2012).

The poplar rust fungi pathogen *Melampsora larici-populina* causes huge yield and ecological loss in poplar trees due to premature leaf loss, and late sprouting after infection (Deecke and Fladung 2021). So far knowledge of the laboratory cultivation of the rust fungi is limited. Difficulties in deciding or formulating a laboratory culturing medium for *M. larici-populina* have occurred. Such difficulties in the laboratory cultivation of the pathogen restrict the research. Similarly, many forest insect pests are either quarantined or restricted from transportation (Steenackers et al. 1996). Moreover, insects like the gypsy moths (*Lymantria dispar*) are difficult to cultivate, the work is laborious as well as tough (Myers et al. 1998). To overcome such hurdles, multidisciplinary collaborations could be a solution. This will help to control the serious pests and also one could carry out the desired study to develop control measures like dsRNA pesticides.

### Inconsistency in RNAi mechanism

RNAi efficiency can vary between insect pests and fungal pathogens, between different orders and.between members of the same pest and pathogen order (Singh et al. 2017; Šečić and Kogel 2021). Even with the same gene, different genes, and areas of the same gene, expression of the gene at different cellular compartments significantly affect the efficiency of the dsRNA (Camargo et al. 2015; Ulrich et al. 2015). Lepidoptera insects are one of the most insensitive insects, while coleopterans are more susceptible to RNAi (Cooper et al. 2019; Santos et al. 2021), thus several insects of Coleoptera showed higher sensitivity towards dsRNA and provided higher possibilities of dsRNA mediated fungal pathogen and insect pest control (Santos et al. 2021).

Various studies have demonstrated that the species from the order Lepidoptera, Diptera, Hemiptera, Coleoptera, and Orthoptera contain nuclease activity in the saliva, midgut, and hemolymph which affects the exogenous dsRNA uptake and reduces the RNAi pesticidal activity (Prentice et al. 2019). Moreover, some fungal species like *Saccharomyces cerevisiae* are missing noticeable homologs of *Ago*, *Dcr*, and *RdRp* which is vital to process exogenously supplied dsRNA and to carry out RNAi silencing (Drinnenberg Ines et al. 2009). A thorough genomic analysis could help in planning and designing laboratory experiments. Furthermore, according to an evolutionary study on different species of the division *Basidiomycota* by Hu et al. (Hu et al. 2013), *M. larici-populina* carries particular and sole domains of the two important genes, *Dcr* and *Ago* needed for RNAi. However, through phylogenetic analysis, authors claim that the poplar rust pathogen has lost the function of both genes. The RNAi-mediated functional analysis of these genes to examine the roles could be of interest.

Uptake of external dsRNA, transport through the gut environment, the solidity of the molecule in the digestive system against high pH, and enzymatic degradation are the most important facts that affect dsRNA efficiency (Guan et al. 2018; Garcia et al. 2017).

### Uptake and further processing of dsRNA

Uptake of artificial or exogenous dsRNA by the cells refers to environmental RNAi that initiate the gene silencing, while the transfer of RNAi signals from one cell to another or tissues within the branched mass of the fungi and body of an insect is recognized as systemic RNAi (Whangbo and Hunter 2008; Huvenne and Smagghe 2010). The presence of both environmental and systematic RNAi is vital in fungal pathogens as well as insect pests (Joga et al. 2016). The Sid-1 channel protein and the scavenger receptor-mediated endocytosis are the two pathways that have been intensively described in insects (Cappelle et al. 2016; Wynant et al. 2014b). Therefore, having one of the active dsRNA uptake pathways is necessary to design RNAi-mediated biocontrol, while the lack of any important protein for dsRNA functioning makes an insect (for example, Lepidopterans) makes less efficient to RNAi (Joga et al. 2016).

The RdRp enzyme carries the primer-independent mechanism, and involve in synthesizing siRNA, and extends the silencing effect important for the synthesis of secondary siRNA is present in many fungi (Cogoni and Macino 1999). On the other hand, direct homologs of RdRp have not been yet observed in the genome of insects, still, insects carry the supporting molecules that work as RdRp to complete the amplification vital for gene silencing (Joga et al. 2016). It is yet to be discovered to what extent the midgut region of the insects acts as a physiological barrier in dsRNA delivery (Christiaens et al. 2020). The epithelial cells, perimicrovillar membrane (PM) of the insect gut play a critical role in the success of RNAi-mediated pest control since the membrane penetrates minerals, vitamins, and cellular molecules (Lehane 1997; Walski et al. 2014).

In principle, the dsRNA is more stable than the single-stranded RNA (ssRNA) and is supposed to penetrate easily through the epithelial cells, PM to trigger the RNAi (Varkouhi et al. 2011). Nevertheless, the nucleases enzyme is present in the salivary and gut regions restricting the RNAi capability by degrading the dsRNA (Christensen et al. 2013). Similar observations have been reported in the pea aphid, *Acyrthosiphon pisum* (Christiaens et al. 2014), the pest desert locust, *Schistocerca gregaria* (Wynant et al. 2014b), and southern green stinkbug *Nezara viridula* (Sharma et al. 2021). The high alkaline pH in the gut of some insects from order lepidopterans plays a crucial role as a barrier of dsRNA resulting in low or no efficient RNAi-mediated gene silencing, in comparison to high acidic pH in coleopterans that shows efficient sensitivity to RNAi (Kolliopoulou et al. 2017).

In fungi, dsRNA needs to be stable in the respective environment and move across the polysaccharide, polycarbohydrates, and glycoproteins of the hyphal wall, and lipid and protein-containing cell membrane (Kang et al. 2018; Athanasopoulos et al. 2019). In fungi, ssDNA, RNA, dsDNA, and dsRNA mycoviruses were already identified (Balabanova et al. 2012). The presence of specific dsRNA degrading nucleases causing trouble for RNAi mechanisms in fungi is not reported, yet the presence of dsRNA-specific nucleases in fungi cannot be denied (Šečić and Kogel 2021). Therefore, the study of these barriers is vital in the targeted fungi pathogen and insect pest.

### dsRNA concentration, size, and formulation

The required length and concentration of dsRNA immensely differ within different insect pests which play a significant role in RNAi success (Bolognesi et al. 2012). Various studies had shown that the required length of dsRNA is depended on the respective insect pest as well as the fungal pathogen. In the red flour beetle, *Tribolium castaneum* a 60 bp long dsRNA showed 70% gene silencing, while 30 bp resulted in only 30% gene silencing. Another study reported that the 140–150 bp dsRNA induces the RNAi. Furthermore, to reduce the sequence homology of the designed dsRNA to off-target effect and non-target species, a short dsRNA is considered ideal (Huvenne and Smagghe 2010; Miller et al. 2012).

Similarly, the optimum concentration of dsRNA is always needed to be determined for individual forest insect pests and fungi pathogens. In some studies, according to the V-ATPase dsRNA, the efficiency of RNAi gene knockdown was observed in the Western corn rootworm, while many studies claim that for better results, increasing the concentration will not induce the gene silencing effect (Baum et al. 2007; Meyering-Vos and Müller 2007). Moreover, in many cases using multiple dsRNA to target different genes probably showed no or mediocre efficiency of RNAi suggesting contest within the introduced dsRNA while cellular uptake (Miller et al. 2012; Barik 2006).

### Candidate gene selection for dsRNA

Successful RNAi-mediated gene silencing depended on the selection of a target gene, the target region within those genes, and its expression in the early developmental stage of the insects (Romeis and Widmer 2020). Selecting a gene with a high transcription rate that translates at the early stage of the insect life, and reduction of the gene transcript should cause mortality in the desired insect pest and forest pathogen vital (Cooper et al. 2020). Screening of several putative genes and the developmental stage-specific expression of these core genes is needed to evaluate for successful applications of RNAi vaccine for selected insect pests and fungi pathogens (Nambiar-Veetil et al. 2011). The genome and transcriptome studies of the particulate pest and pathogen evaluate the expressional changes in different life stages (Oppert and Perkin 2019). An insect microbiome study that could affect the provided dsRNA is also considered optimal for better RNAi vaccination strategies (Xu et al. 2021).

Studies have shown the cross-kingdom communication and exchange of small RNAs (sRNAs) between fungi and plants that could knock down the immune response in plants and virulence genes in fungi (Cai et al. 2018; Ren et al. 2019; Weiberg et al. 2015). Thus, targeting fungal virulence genes for dsRNA design is essential for successful RNAi gene silencing and pest control.

### Species-specific dsRNA

To initiate the RNAi mechanism, a dsRNA with 80% and more sequence complementary is needed, revealed through mutagenesis analysis. Moreover, a dsRNA that holds a homology of ≥ 16 bp or > 26 bp regions with two mismatches also activates the RNAi-mediated gene knockdown (Chen et al. 2021). Similarly, in the species of plant sap-feeding hemipteran pests, the higher sequence homology is very important to trigger RNAi-mediated gene silencing (Arora et al. 2021).

The difference in the transcription abundance of a gene has been observed among developing life stages suggesting the concentration of dsRNA is also required to adjust regarding the candidate dsRNA gene. Hence, a species-specific concentration and/or overdose (if required) of the dsRNA according to the abundance of nucleases is essential to rigger RNAi-dependent gene silencing (Scott et al. 2013). Most importantly, selecting a non-conserved region of other species or isoforms of the target gene is important for species-specific RNAi-dependent dsRNA and to avoid the non-target effect.

## dsRNA pesticide development for forest insect pests and fungal pathogens

### Use of high-throughput sequencing for off-target and non-target effects

Emerging advancements in high-throughput sequencing (HTS) technologies have made available the genome and transcriptomes of many forest insect pests and fungi pathogens. The reducing sequencing cast of advanced sequencing techniques can play an important role in species-specific dsRNA design (Rodrigues et al. 2017a). Moreover, continuously improvised genome-wide screening methods and utilization of available data play a vital role to reduce off-target and non-target effects as well as more intensive results are also needed for accuracy (Wang et al. 2011; Knorr et al. 2018). In RNAi, the off-target effects are referred to as when a dsRNA influence the expression of genes other than the targeted gene causing unnecessary modifications (Birmingham et al. 2006). In contrast, to avoid any adverse consequences of designed dsRNA on species like humans, farm animals, and beneficial species mentioned as a non-target effect (Hollowell and Rieske 2022). Many previous studies with well-designed dsRNA including targeted γ-Tubulin in *Drosophila*, and *CYP* genes of the Tobacco hornworm (*Manduca sexta*) showed high species-specificity (Whyard et al. 2009; Kumar et al. 2012). To achieve such prominent results, we need to take advantage of HTS. Fungi require *Ago, RdRp,* and *Dcl* to carry out the RNAi, therefore identification and copy number of these proteins should be conducted using bioinformatics tools before the application of dsRNA (Laurie et al. 2008).

In a recent study on the first sprayable dsRNA pesticide against Colorado Potato Beetle, authors have used genome and transcriptome sequences to avoid off- and non- target effects to develop the product, Ledprona. A low-cost sRNA sequencing of the plant, insect pest, or fungi pathogen where dsRNA was applied will assure the guarantee of synthesized dsRNA pesticide by genomic mapping (Rodrigues et al. 2021). Moreover, transcriptome analysis of forest insect pests and fungal pathogens will help to select a potential candidate gene to develop dsRNA as previously described (Ulrich et al. 2015). Even though an exponential reduction has been in the price for sequencing, genome, and transcriptome information on many devastating forest pests and pathogens is still missing, including Oak processionary (*Thaumetopoea processionea*) (Table 2).

**Table 2:** Overview of genome and transcriptomics studies conducted with forest insect pests and fungi pathogens. The list has been prepared after searching the name of each pest and pathogen in the NCBI gene database bank. The genome and transcriptome accession numbers were collected according to the NCBI gene database bank

Common name	Scientific name	Genome/assembly accession	Transcriptome accession
European gypsy moth	*Lymantria dispar dispar L*	PRJNA694036 PRJNA504524	PRJNA789495
Hemlock woolly adelgid	*Adelges tsugae*	-	PRJNA242203
Redbay ambrosia beetle	*Xyleborus glabratus*	-	PRJNA495609
Emerald ash borer	*Agrilus planipennis*	PRJNA343475, PRJNA339096, PRJNA230921	PRJNA286481, PRJNA271706, PRJNA263193, PRJNA222581, PRJNA79619
Asian longhorn beetle	*Anoplophora glabripennis*	PRJNA348318, PRJNA274806, PRJEB3278, PRJEB434, PRJNA17217	PRJNA691113, PRJNA299040, PRJNA281943, PRJNA274806
Winter moth	*Operophtera brumata*	PRJNA293072	-
Polyphagous shot-hole borer	*Euwallacea fornicatus*	PRJNA790378	PRJNA260703
Woodwasp	*Sirex noctilio*	PRJNA507701, PRJNA513942	PRJNA204857, PRJNA252354, PRJNA530843
*Southern pine beetle* (SPB)	*Dendroctonus frontalis*	PRJNA493650	PRJNA584450, PRJNA584449, PRJNA584448
Spruce budworm	*Choristoneura fumiferana*	PRJNA453333	PRJNA526661
Mountain pine beetle	*Dendroctonus ponderosae*	PRJNA638278, PRJNA638274, PRJNA360270, PRJNA269763	PRJNA37293, PRJNA157397, PRJNA178770, PRJNA189795
Red turpentine beetle	*Dendroctonus valens*	PRJNA780393	PRJNA609406
Spruce bark beetle	*Ips typographus*	PRJNA286539, PRJNA671615,	PRJNA178930, PRJNA602798, PRJNA679450
Mediterranean pine beetle	*Orthotomicus erosus*	PRJNA806059, PRJNA782296	
Cockchafers	*Melolontha spp*	PRJEB50970	PRJEB50970
-	*Cinara cupressivora*	PRJNA295713	PRJNA295713
-	*Heteropsylla cubana*	PRJNA483576	PRJNA272248
Oak lace bug	*Corythucha arcuata*	PRJNA295691	PRJNA295691
-	*Leptocybe invasa*	PRJNA305347	PRJNA305347
-	*Sirex noctilio*	-	PRJNA204857
Nun moth	*Lymantria monacha*	PRJEB42031, PRJEB42030	PRJNA230562
Eastern spruce budworm	*Choristoneura fumiferana*	PRJNA453333	-
Western spruce budworm	*Choristoneura occidentalis*	PRJNA453223, PRJNA4532220	-
Jack-pine budworm	*Choristoneura pinus*	PRJNA495524	PRJNA495524
Oak-leaf roller	*Tortrix viridana*	-	PRJEB1903
-	*Thaumetopoea pityocampa*	PRJNA703101, PRJNA593925, PRJNA344465	PRJNA268248, PRJNA232643
Balsam Woolly Adelgid	*Adelges piceae*	PRJNA574620	-
Willow Leaf Beetle	*Plagiodera versicolora*	PRJNA700218, PRJNA497053	-
Oak Wilt	*Ceratocystis fagacearum*	PRJNA677221, PRJNA342331	PRJNA677222
Neonectria canker of fir	*Neonectria neomacrospora*	PRJEB41540	-
Pine-tree lappet moth	*Dendrolimus pini*	PRJNA267874	PRJNA267874
Large pine weevil	*Hylobius abietis*	PRJNA435679	-
Horse-chestnut leaf miner,	*Cameraria ohridella*	-	PRJNA267869
Elm zigzag sawfly	*Aproceros leucopoda*	-	PRJNA252225
Citrus longhorn beetle	*Anoplophora chinensis*	PRJNA311893	PRJNA400573
Fungi pathogens			
Chestnut blight	*Cryphonectria parasitica* *(Murrill) Barr*	PRJNA644891 PRJNA492223	PRJNA642399 PRJNA588887 PRJNA258639
White pine blister rust	*Cronartium ribicola*	PRJNA190829 PRJNA352055	PRJNA261951 PRJNA222839
Phytophthora dieback	*Phytophthora cinnamomi*	PRJNA675400	PRJNA675400 PRJEB8451
Port-Orford-cedar root disease	*Phytophthora lateralis*	PRJNA190827	-
Beech bark disease	*Nectria coccinea var. faginata*	• PRJNA593731	• -
Sudden oak death	*Phytophthora ramorum*	PRJNA738483	-
Dutch elm disease	*Ophiostoma ulmi*	PRJNA173023	-
Oak Root Fungus	*Armillaria Root Rot*	PRJNA629806 PRJNA390997	-
Ash dieback	*Hymenoscyphus fraxineus*	PRJEB6546	PRJEB7998
Root rot pathogen	*Heterobasidion irregulare*	PRJEB8921 PRJNA245138 PRJNA46703	-
Poplar rust fungus	*Melampsora larici-populina*	PRJNA749283	PRJNA749283
Rhizoctonia Root Rot	*Rhizoctonia sp.*	PRJNA778030	PRJNA744257
Charcoal cankers	*Biscogniauxia mediterranea*	PRJNA727443 PRJNA571054	PRJNA570004 PRJNA567201
Red band needle blight	*Dothistroma septosporum or* *Dothistroma pini*	PRJNA381823 PRJNA74753	PRJNA584708 PRJNA584643
Pitch canker	*Fusarium circinatum*	PRJNA719068 PRJNA716280	PRJNA666978
Pine-needle blight	*Lecanosticta acicola*	PRJNA399865 PRJNA390145 PRJNA212329	-
Dothistroma needle blight	*Mycosphaerella pini*	PRJNA381823, PRJNA74753	PRJNA584708, PRJNA584643,
Butternut canker	*Ophiognomonia clavigignenti* *juglandacearum*	PRJNA434132	-
Diplodia tip blight	*Sphaeropsis sapinea / Diplodia sapinea*	PRJNA242796, PRJNA215898	-
Verticillium wilt	*Verticillium dahliae*	PRJNA725559, PRJNA725553, PRJNA692217	PRJNA587035, PRJNA735544
Fusarium Head Blight	*Fusarium spp.*	PRJNA389173, PRJNA361522	-
Gray mold	*Botrytis cinerea*	PRJNA700674, PRJNA624742, PRJNA525902	PRJNA687991
Snow Blight	*Phacidium infestans*	PRJNA695287	-
Powdery Mildews	*Microsphaera alphitoides*	PRJNA593204	-
Septoria Canker	*Sphaerulina musiva*	PRJNA584852, PRJNA584707,	PRJNA584852, PRJNA584707
Oak powdery mildew	*Erysiphe* sp	PRJNA738036, PRJNA737954	PRJNA583961, PRJNA583960
Rhizina root disease	*Rhizina undulata*	PRJNA372875, PRJNA333148	PRJNA372875
Beech tar crust	*Biscogniauxia nummularia*	PRJNA331563,	PRJNA650798, PRJNA650797
	*Botryosphaeria dothidea*	PRJNA782772, PRJNA782403, PRJNA660898	PRJNA450131, PRJNA431921
Sphaeropsis blight	*Diplodia pinea*	PRJNA242796, PRJNA215898	*-*
Hypoxylon canker	*Entoleuca mammata*	PRJNA463999, PRJNA347216	PRJNA463999
European larch canker	*Lachnellula willkommii*	PRJNA412379	*-*
Passalora needle blight	*Passalora sequoiae*	PRJNA595369	*-*
-	*Phytophthora pluvialis*	PRJNA316743, PRJNA290686, PRJNA290685	*-*
Diplodia Tip Blight	*Diplodia pinea*	PRJNA242796, PRJNA215898	-
-	*Cryphonectria radicalis*	PRJNA379967, PRJNA379946, PRJNA279979	-
Brunchorstia disease	*Gremmeniella abietina*	PRJNA463998, PRJNA347218, PRJNA347217	PRJNA713183, PRJNA463998

### Nanoparticle-mediated dsRNA delivery

Enveloping of dsRNA using organic and inorganic particles typically one to a few hundred in size called nanoparticles, helps in molecular transport. Various studies have successfully shown enhanced RNAi efficacy when dsRNA is delivered using nano-complexes. The dsRNA coated with nanomaterial showed improved dsRNA absorbance, stability, protection against nucleases, and improved cellular uptake of the dsRNA (Yan et al. 2020, 2021). For dsRNA or siRNA delivery in the targeted organism using chitosan-derived nanoparticles has been substantially used due to their non-toxic and biodegradable properties (Zhang et al. 2015). The dsRNA intended to silence the *CHITIN SYNTHASE B* gene in the beet armyworm (*Spodoptera exigua*) protected against gut nucleases and showed increased mortality when supplied through guanylate polymers (Christiaens et al. 2018). Control of many insect pests was reported using Nanoparticle encapsulation proving its efficiency. The nanoparticle-mediated dsRNA delivery could also be used with petiole absorption and trunk injection considered a GMO-free approach (Joga et al. 2021). Moreover, the nanocarrier-mediated dsRNA transfer through spray has already displayed its success. Yan and co-workers have described the prospectus of nanoparticle-based dsRNA delivery (Yan et al. 2021).

However, nanoparticles also could have harmful effects on human and animal health and on the environment. For instance, inhaled nanoparticles in the body may result in lung inflammation and heart problems (Ec.europa.eu n.d). Also, nanoparticles are able to cross the blood–brain barrier, which makes them extremely dangerous as a carrier to deliver toxic substances directly to the brain (Olivier et al. 1999). The effects of nanoparticles on the environment have been poorly investigated so far (Colvin 2003). However, because several effects of nanoparticles have been identified for humans, it is likely that also numerous effects exist on plant and animal species other than humans.

### Biosafety of dsRNA

The RNAi-mediated dsRNA fungi- and pesticide could reduce the off- and non- target effects in the ecology which is difficult with traditional chemical pesticides that adversely effects on respective ecology as well as the environment. However, dsRNA pesticides might need to consider new ecological and environmental evaluation obstacles. The exogenous dsRNA degrades rapidly in the environment when applied to forest trees to manage fungal pathogens and insect pests, hence it should not consider a genetically modified product. Nevertheless, a biosafety analysis of dsRNA pesticides before their commercialization is very important (Shew et al. 2017; Arpaia et al. 2021).

In the era of genomics, the bioinformatic assessment will play a crucial role in the species-specific objective of the designed dsRNA. Finally, the consequences of exogenous dsRNA on the forest ecology will be needed to evaluate precisely soil, other plants, and associated benefits as well as symbiotic microorganisms.

We agree with the author Joga and coworkers (Joga et al. 2021) that the various regulatory authorities need to consider different aspects of the dsRNA methods. It is possible that the dsRNA treated and used organism can be subject to the evaluation of environmental stress and biosafety including the active ingredient, environmental fate of dsRNA, and their impact on non-target organisms. Considerably, targeted dsRNA to function as RNAi pesticides are clearly present within the selected organisms. Hence, dsRNA could be considered most likely a promising and secure alternative to traditional chemical pesticides.

### Genetically modified organism–free approach

Earlier, RNAi techniques have been traditionally established using recombinant plants, viruses (virus-induced gene silencing), and *Agrobacterium tumefaciens*-mediated transformation carrying dsRNA for a targeted gene well known as host-induced gene silencing (HIGS) (Baulcombe 2004). However, the utilization of genetically modified organisms (GMOs) has increased public and scientific worry and discussions. Therefore, the substitute method that evades the use of recombinant genes or proteins is preferred widely. More interest in GMO-free approaches without using recombinant viruses or transgenes but exogenous direct administration of dsRNA that can initiate the RNAi-mediated gene silencing aroused (Dalakouras et al. 2020). According to the reports, plants served with exogenous dsRNA are not GMOs (Taning et al. 2020) since the dsRNA could be directly provided through the spray, trunk injection, root soaking, etc. (Fig. 2). Therefore, within a short time, the great potential of RNAi against outbreak-causing pests and pathogens was studied (Joga et al. 2021).

### Mycorrhizal mediated dsRNA delivery

The fungal-induced gene silencing (FIGS) method to deliver dsRNA and enhance the effect of RNAi is already been tested to control forest insect pests (Van Ekert et al. 2014; Mysore et al. 2019). Using *Saccharomyces cerevisiae* and entomopathogenic fungi carrying targeted dsRNA to increase the RNAi regulation in insect pests (Mysore et al. 2019; Hu and Wu 2016). Studies on dipteran insects showed significant mortality and delayed larval development when pellets of dsRNA-transformed yeast were provided through diet (Van Ekert et al. 2014; Mysore et al. 2019). Similarly, the *S. cerevisiae* expressing dsRNA targeting the *Y-TUBULIN 23C* (*YTUB23C*) gene showed reduced larval survival, and the egg-production rate in *Drosophila suzukii* when provided orally (Murphy et al. 2016). However, the approaches are related to GM and might face a huge worry among the public.

Here we propose a new ‘mycorrhiza-mediated dsRNA’ delivery method to control forest insect pests as well as fungi pathogens. The most critical step for the success of the suggested method is the uptake of dsRNA by the fungal hyphae, formed of chitin, polysaccharides, glycoproteins, and plasma membrane with lipids and proteins (Kang et al. 2018). Favorably, fungi can directly consume dsRNA which is detected in various fungi without any obstruct from the cell wall and cell membrane (Wytinck et al. 2020a). The liquid culture of fungi co-incubating with fluorescently-labeled dsRNA was able to uptake the dsRNA, observed in *Botrytis cinerea* spores growing on agar medium and in protoplasts (Wang et al. 2016). The dsRNA targeting ergosterol biosynthesis genes was sprayed on *F. graminearum* to reduce the infection (Koch et al. 2016). Additionally, the various dsRNAs were subjected to the liquid cultures of *Sclerotinia sclerotiorumi* to examine the uptake and silencing efficiency (McLoughlin et al. 2018). A remarkable study shows that clathrin-mediated endocytosis (CME) is responsible for the exogenous dsRNA uptake *S. sclerotiorum* (Wytinck et al. 2020b). All the studies show, the direct uptake of dsRNA by the fungi when co-incubated together which is considered a GMO-free approach.

Further, the plants could be inoculated with mycorrhiza cultures carrying dsRNA targeting a specific gene against the forest pest and pathogens. Nutrition transfer from both, plants and mycorrhiza is a key feature of the symbiosis association. Mycorrhiza receives carbon synthesized by the plants through photosynthesis while plants acquire water and minerals. The nutrition exchange is carryout through the fungal and plant root cell walls (Ferrol et al. 2002). In the process of symbiosis and to carry out nutrition exchange, microbes alter the plant cell walls causing beneficial invasion according to needs (Oelmüller 2018). Besides nutrition, the symbiotic association also could exchange nucleic acids between both partners. A study shows the plant nucleic acid stresses within the *Rhizophagus irregularis* mycorrhiza genome suggest horizontal gene transfer through endosymbiosis (Li et al. 2018a). During the mycorrhizal interaction, the effector protein of SP7 from *R. irregularis* binds to the transcriptional factor ERF19 of the host (Van Heijden et al. 2015).

**Fig. 3:**
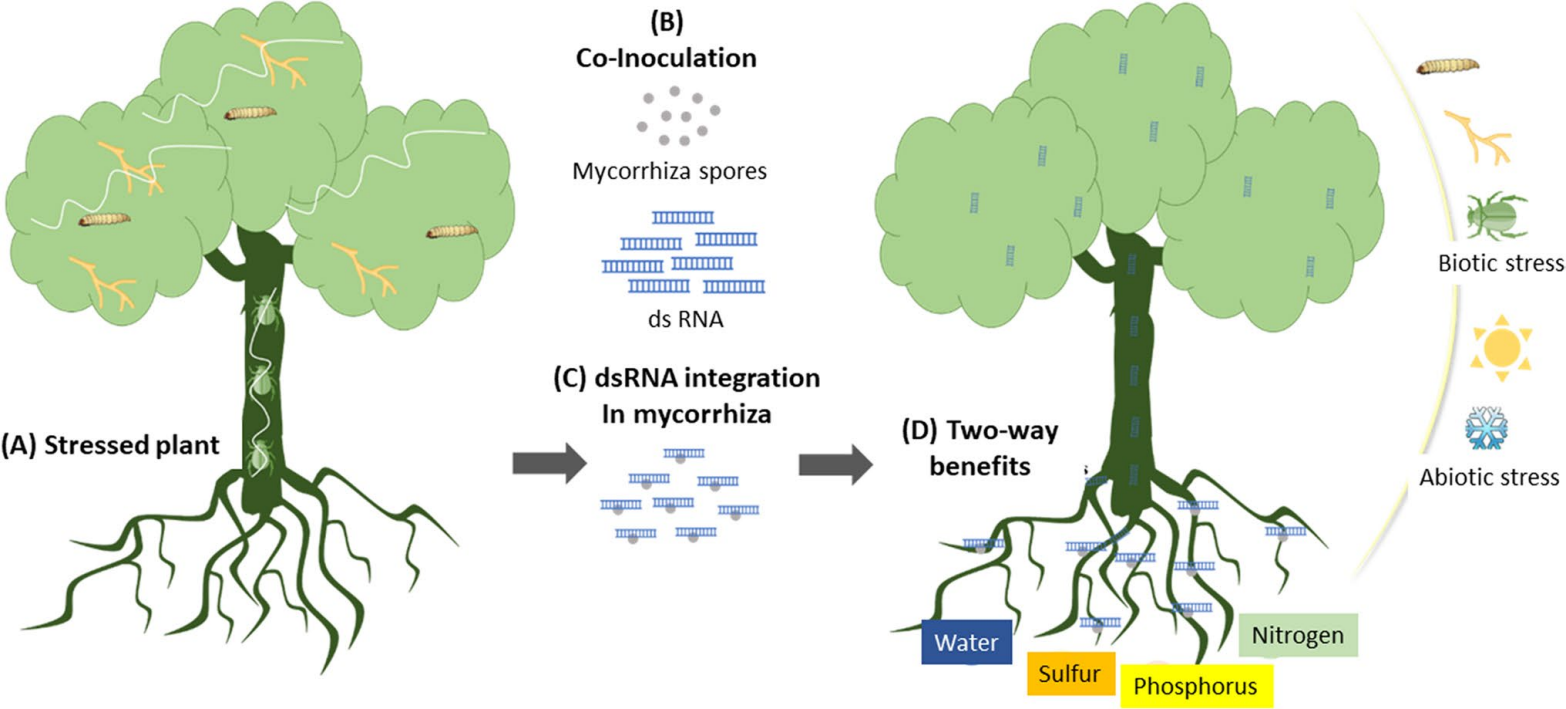
Mycorrhiza mediated dsRNA delivery, the two-way benefit for woody and forest plants: A) various biotic and abiotic means cause stress to the forest trees, B) co-inoculation (a non-GMO approach) of mycorrhiza spores with desired dsRNA, C) lead to the integration. D) The applied dsRNA and mycorrhiza lead to the two-way benefit for the plants including dsRNA vaccination against pests and pathogens, followed by traditional mycorrhiza symbiosis

Similarly, like nutrition and nucleic acids, mycorrhiza could also supply the desired dsRNA to the plants. Once the dsRNA reaches the cortex of the roots, we assume it further could transport to the stem and leaves through the phloem, similarly to nutrition and nucleic acids (Fig. 3). The suggested mycorrhiza-mediated dsRNA delivery approach could serve a two-way benefit to the forest trees including traditional benefits of the mycorrhiza followed by dsRNA pesticide and fungicide. Applications of mycorrhiza have been integrated into plant protection for hundreds of years. Instead of common yeast and/or entomopathogenic fungi, we suggest using mycorrhiza carrying the desired dsRNA. Application of such mycorrhiza at the bottom of trees may enhance the health of the trees by providing nutrients and the dsRNA could protect them from insect pests and fungal pathogens.

Similar efforts have been made with symbiotic bacteria that carry dsRNA for the desired gene in the insect. The phenotypes in the insects were observed when *Rhodococcus rhodnii*, an endogenous plant symbiont carrying a dsRNA targeting multiple genes was fed to the insects (Whitten et al. 1825). Indeed, it is a preliminary idea that needs laboratory experiments to understand and confirm the suggested method. We agree that it is just a preliminary idea that needs a significant amount of work considering its environmental release, ecological pressure and the possibility of alteration in the genome of soil microbial community changes, and the regulations. We have addressed possible challenges and solutions in the above sections.

Advantage of the reducing genome sequencing costs will help to design a species-specific dsRNA that will not affect mycorrhiza. Integration of dsRNA within the mycorrhiza could be possible through co-incubation. Furthermore, plants would be inoculated with the mycorrhiza carrying dsRNA targeting a vital gene of the pest or pathogen. Such a target-specific approach could play an effective or at least alternative role in forest tree protection against pathogens and pests.

## Conclusions

Although the cost of NGS techniques is reducing significantly, the genomic and sequencing knowledge of many important forest insect pests and the fungal pathogen is still missing. Developing RNAi-mediated forest pest and pathogen control strategies requires support from sequencing technology. Many forest insect pests have been tested against GMO-free dsRNA in the laboratory, however, their field or greenhouse applications still needed to be tested. Most importantly, we need to solve challenges like the selection of superior target genes in pathogens and pests, non-target effects), stability of dsRNA, formation of small RNA molecules, and organism-specific cellular barriers including nucleases to develop dsRNA-mediated pathogen and pest control. Furthermore, the dsRNA manufacturing cost is considerably high, however, advances in large-scale COVID-19 (SARS-CoV2) vaccine production could be helpful to produce dsRNA-based fungicides and pesticides at affordable costs (Taning et al. 2020).

Spray-, petiole absorption-, and trunk injection-dsRNA delivering strategies might not be optimum to control the forest insect pest and fungi but they will enable to study of the synthesized dsRNA in the field and greenhouse. The proposed, novel mycorrhiza-mediated dsRNA method could make a revolution in RNAi-mediated forest protection.

## Supplementary Information

Below is the link to the electronic supplementary material.

Supplementary file1 (DOCX 55 KB)

## Data Availability

All the data and material has submitted.
