# Supplementary material for: Double-stranded RNA (dsRNA) technology to control forest insect pests and fungal pathogens: challenges and opportunities
Source: Funct Integr Genomics. 2023 May 27;23(2):185. doi: 10.1007/s10142-023-01107-y (PMC10220346; doi:10.1007/s10142-023-01107-y)
Supplement: Supplementary file 1 — Supplementary file1 (DOCX 55 KB) [file 10142_2023_1107_MOESM1_ESM.docx]

**Table S1:** The records on forest fungal pathogens and/or diseases that have and had caused outbreaks in Oak, Conifer, Birch, Poplar, Ash, Beech, Maple, Elm, and Acer trees using the keywords “forest pathogens” in Google Scholar search. The common name of the pathogens, scientific name, host trees, geographical distribution, and references are summarized. Species that have caused outbreaks are represented with the BOLD letter. NA: North America; EU: Europe; USA: United States of America; NZ: New Zealand; UK: United Kingdom.

| **Common name** | **Scientific name** | **Host tree** | **Geographic range** | **References** | **Outbreak** |
| --- | --- | --- | --- | --- | --- |
| **Chestnut blight** | ***Cryphonectria parasitica*** | **Beech, Chestnut** | **Asia, NA, EU** | **Liberato JR & Robin C (2006) Chestnut blight (*Cryphonectria parasitica*) Updated on 7/28/2016 8:31:46 AM Available online: PaDIL** | [**http://www.padil.gov.au**](http://www.padil.gov.au/)  [**https://www.appsnet.org/Publications/potm/pdf/Mar13.pdf**](https://www.appsnet.org/Publications/potm/pdf/Mar13.pdf) |
| **White pine blister rust** | ***Cronartium ribicola*** | **Pine** | **Asia, NA** | [**https://www.cabi.org/isc/datasheet/16154**](https://www.cabi.org/isc/datasheet/16154)  [**https://doi.org/10.5962/bhl.title.51507**](https://doi.org/10.5962/bhl.title.51507)  [**https://doi.org/10.1111/j.1439-0329.2006.00432.x**](https://doi.org/10.1111/j.1439-0329.2006.00432.x) | [**https://www.apsnet.org/edcenter/disandpath/fungalbasidio/pdlessons/Pages/WhitePine.aspx**](https://www.apsnet.org/edcenter/disandpath/fungalbasidio/pdlessons/Pages/WhitePine.aspx)  **https://www.frontiersin.org/articles/10.3389/ffgc.2021.765871/full** |
| **Phytophthora dieback** | ***Phytophthora cinnamomi*** | **Chestnut, Oak, Pine** | **EU, NA, Portugal** | [**http://www.issg.org/database/species/ecology.asp?si=143&fr=1&sts&lang=TC**](http://www.issg.org/database/species/ecology.asp?si=143&fr=1&sts&lang=TC)  [**https://doi.org/10.1111/jph.12931**](https://doi.org/10.1111/jph.12931) | [**http://www.issg.org/database/species/ecology.asp?si=143&fr=1&sts&lang=TC**](http://www.issg.org/database/species/ecology.asp?si=143&fr=1&sts&lang=TC)  [**https://doi.org/10.1111/jph.12931**](https://doi.org/10.1111/jph.12931) |
| **Port-Orford-cedar root disease** | ***Phytophthora lateralis*** | **Pine** | **NA, EU** | [**http://www.issg.org/database/species/ecology.asp?si=143&fr=1&sts&lang=TC**](http://www.issg.org/database/species/ecology.asp?si=143&fr=1&sts&lang=TC)  [**https://doi.org/10.1111/jph.12931**](https://doi.org/10.1111/jph.12931) | [**http://www.issg.org/database/species/ecology.asp?si=143&fr=1&sts&lang=TC**](http://www.issg.org/database/species/ecology.asp?si=143&fr=1&sts&lang=TC)  [**https://doi.org/10.1111/jph.12931**](https://doi.org/10.1111/jph.12931) |
| **Beech bark disease** | ***Nectria coccinea var. faginata*** | **American beech** | **NA** | [**https://www.forestresearch.gov.uk/tools-and-resources/fthr/pest-and-disease-resources/phytophthora-lateralis/**](https://www.forestresearch.gov.uk/tools-and-resources/fthr/pest-and-disease-resources/phytophthora-lateralis/)  [**http://download.ceris.purdue.edu/file/2146**](http://download.ceris.purdue.edu/file/2146) | [**https://esajournals.onlinelibrary.wiley.com/doi/10.1890/15-1176**](https://esajournals.onlinelibrary.wiley.com/doi/10.1890/15-1176) |
| **Sudden oak death** | ***Phytophthora ramorum*** | **Aak** | **NA, EU** | [**https://doi.org/10.1080/00275514.1941.12020808**](https://doi.org/10.1080/00275514.1941.12020808)  [**https://hal.archives-ouvertes.fr/hal-00882224/document**](https://hal.archives-ouvertes.fr/hal-00882224/document) | [**https://doi.org/10.1080/00275514.1941.12020808**](https://doi.org/10.1080/00275514.1941.12020808)  [**https://hal.archives-ouvertes.fr/hal-00882224/document**](https://hal.archives-ouvertes.fr/hal-00882224/document) |
| **Dutch elm disease** | ***Ophiostoma ulmi*** | **Elm trees** | **EU, NA** | [**https://www.efsa.europa.eu/en/press/news/110628**](https://www.efsa.europa.eu/en/press/news/110628)  [**https://www.cell.com/trends/microbiology/fulltext/S0966-842X(11)00227-7**](https://www.cell.com/trends/microbiology/fulltext/S0966-842X(11)00227-7) | **https://archive.org/details/dutchelmdiseaseg00clin/page/n3/mode/2up** |
| - | *Fusarium euwallacea* | More than 400 host species | USA, NA | - https://doi.org/10.3852/13-066 | - **-** |
| **Oak Root Fungus** | ***Armillaria Root Rot*** | **Oak** | **USA** | [**http://ipm.illinois.edu/diseases/series600/rpd602/**](http://ipm.illinois.edu/diseases/series600/rpd602/)  **https://www.jstor.org/stable/42601640** | [**http://ipm.illinois.edu/diseases/series600/rpd602/**](http://ipm.illinois.edu/diseases/series600/rpd602/)  **https://www.jstor.org/stable/42601640** |
| **Swiss needle cast (SNC)** | ***Phaeocryptopus gaeumannii*** | **Douglas-fi r** | **NA** | **https://bsppjournals.onlinelibrary.wiley.com/doi/10.5197/j.2044-0588.2014.029.019** | **https://bsppjournals.onlinelibrary.wiley.com/doi/10.5197/j.2044-0588.2014.029.019** |
| **Ash dieback** | ***Hymenoscyphus fraxineus*** | **Ash** | **EU, UK** | **https://nph.onlinelibrary.wiley.com/doi/10.1002/ppp3.11** | **https://nph.onlinelibrary.wiley.com/doi/10.1002/ppp3.11** |
| **Spruce needle rust** | ***Chrysomyxa ledicola*** | **Spruce** | **USA** | **https://www.apsnet.org/publications/PlantDisease/BackIssues/Documents/1986Abstracts/PD_70_801d.htm** | **https://www.apsnet.org/publications/PlantDisease/BackIssues/Documents/1986Abstracts/PD_70_801d.htm** |
| **Root rotting fungi** | ***Heterobasidion irregulare*** | **Spruce** | **EU** | **Gonthier P., R. Warner, G. Nicolotti, A. Mazzaglia and M. Garbelotto, 2004. Pathogen introduction as a collateral effect of military activity. Mycological Research 108, 468–470.**  [**https://doi.org/10.1038/s41598-020-62521-x**](https://doi.org/10.1038/s41598-020-62521-x) | **https://www.mdpi.com/1999-4907/12/1/57/htm** |
| **Poplar rust fungus** | ***Melampsora larici-populina*** | **Poplar** | **Asia, EU, NA** | **https://link.springer.com/article/10.1007/s12600-013-0294-0** | **https://doi.org/10.1007/s11676-015-0021-4** |
| Poplar rust fungus | *Melampsora allii-populina* | Poplar | Asia, EU, NA | https://bsppjournals.onlinelibrary.wiley.com/doi/10.1111/ppa.12426 | - |
| Rhizoctonia Root Rot | *Rhizoctonia* sp. | Pine | NA | https://doi.org/10.1007/978-94-017-2901-7_32 | - |
| **Charcoal cankers** | ***Biscogniauxia mediterranea*** | **Oak** | **NA, EU** | **https://bsppjournals.onlinelibrary.wiley.com/doi/10.1111/j.1365-3059.2005.01297.x** | **https://bsppjournals.onlinelibrary.wiley.com/doi/10.1111/j.1365-3059.2005.01297.x** |
| **Red band needle blight** | ***Dothistroma septosporum* or**  ***Dothistroma pini*** | **Pine** | **Asia, EU** | **New country and regional records of the pine needle blight pathogens**  ***Lecanosticta acicola*, *Dothistroma septosporum* and *Dothistroma pini*** | ***https://doi.org/10.1139/X09-159*** |
| **Pitch canker** | ***Fusarium circinatum*** | **Pine and Douglas fir** | **USA, NA, EU** | **Pitch canker caused by *Fusarium circinatum*-a growing**  **threat to pine plantations and forests worldwide.**  **First report of *Fusarium oxysporum* f. sp. *palmarum* in Texas causing *Fusarium* wilt of Washingtonia robusta**  **https://apsjournals.apsnet.org/doi/abs/10.1094/PD-89-1015A** | [**https://doi.org/10.1094/PD-89-1015A**](https://doi.org/10.1094/PD-89-1015A)  **https://apsjournals.apsnet.org/doi/abs/10.1094/PD-89-1015A** |
| **Pine-needle blight** | ***Lecanosticta acicola*** | **Pine** | **USA, EU** | ***Biodiversity of Lecanosticta pine-needle blight pathogens suggests a Mesoamerican Centre of origin*** | [***https://silvabalcanica.pensoft.net/article/54610/***](https://silvabalcanica.pensoft.net/article/54610/) |
| **Dothistroma needle blight** | ***Mycosphaerella pini*** | **Pine** | **USA, NA, EU** | **Simulating the effects of a climate-change scenario on the geographical range and activity of forest-pathogenic fungi** | **https://doi.org/10.1139/X09-159** |
| **Beech bark disease** | ***Neonectria coccinea*** | **Beech** | **USA, NA, EU** | **Species delimitation for *Neonectria coccinea* group including the causal agents of beech bark**  **disease in Asia, Europe, and North America** | **https://www.tandfonline.com/doi/abs/10.3852/08-165** |
| **Butternut canker** | ***Ophiognomonia clavigignentijuglandacearum*** | **Beech. butternut** | **NA, USA** | **Influence of temperature and humidity on the viability of *Ophiognomonia clavigignenti-juglandacearum* conidia.**  **Renlund, D. W. comp. ed. 1971. Forest pest conditions in Wisconsin, annual report 1971. Wisconsin Department of Natural Resources, Madison, WI. p. 26-28.** | **https://www.biorxiv.org/content/10.1101/820977v1.full** |
| **Diplodia tip blight** | ***Sphaeropsis sapinea*** | **Scots pine and Conifers** | **EU. NA** | **Simulating the effects of a climate-change scenario on the geographical range and**  **activity of forest-pathogenic fungi**  [**https://doi.org/10.1007/s11557-020-01617-0**](https://doi.org/10.1007/s11557-020-01617-0) | ***https://www.frontiersin.org/articles/10.3389/ffgc.2021.655769/full*** |
| **Verticillium wilt** | ***Verticillium dahliae*** | **Over 400 plants** | **Asia, EU, NA** | **Transcriptomic profiles of the smoke tree wilt fungus *Verticillium dahliae* under nutrient starvation stresses** | **https://www.frontiersin.org/articles/10.3389/fpls.2021.632689/full** |
| Poplar tongue gall | *Taphrina johansonii and*  *Taphrina rhisophorus* | Poplar | EU | https://doi.org/10.1099/ijs.0.052712-0 | - |
| **Leaf desiccation of beech** | ***Gloeosporium fagi*** | **European beech *(Fagus sylvatica)*** | **EU** | **Disseccamenti delle foglie di faggio da Gloeosporium fagi (Desm. e Rob.) Westend** | **https://trace.tennessee.edu/utk_gradthes/3290/** |
| **Pitch Canker** | **Fusarium circinatum** | **Pine** | **NA, EU** | **https://pubmed.ncbi.nlm.nih.gov/30786652/** | **https://pubmed.ncbi.nlm.nih.gov/30786652/** |
| **-** | **Fusarium solani** | **Oak** | **NA, EU** | [**https://pubmed.ncbi.nlm.nih.gov/30845451/**](https://pubmed.ncbi.nlm.nih.gov/30845451/) |  |
| **-** | **Fusarium oxysporum** | **Fir** | **NA, EU** | [**https://pubmed.ncbi.nlm.nih.gov/7214223/**](https://pubmed.ncbi.nlm.nih.gov/7214223/) |  |
| **Gray mold** | ***Botrytis cinerea*** | **Spruce, Pine** | **EU** | [**https://www.hindawi.com/journals/isrn/2012/810675/**](https://www.hindawi.com/journals/isrn/2012/810675/)  **Gadgil, P.D. 2005: Fungi on trees and shrubs in New Zealand. Fungi of New Zealand Volume 4. Fungal Diversity Research Series 16: 1-437.** | **https://www.hindawi.com/journals/isrn/2012/810675/** |
|  | *Phomopsis quercella* | Oak | EU | https://doi.org/10.1007/978-3-319-89833-9_10 | - |
|  | *Cytospora intermedia* | Oak | EU | Oskay, Funda & Imal, Bora & Orhan, Fatma & Meşe, Özlem. (2019). Preliminary Results on The Fungi Damaging Pedunculate Oak (Quercus Robur L.) Acorns. | - |
| **Lophodermium Needle Casts** | ***Lophodermium seditiosum*** | **Pine** | **EU** | **https://doi.org/10.3390/f11070718** | **https://doi.org/10.3390/f11070718** |
| **-** | ***Lophodermium macrosporum*** | **Spruce** | **EU** | [**https://agris.fao.org/agris-search/search.do?recordID=US201302608249**](https://agris.fao.org/agris-search/search.do?recordID=US201302608249) | **Hanso, Märt & Drenkhan, Rein. (2007). Retrospective Analysis of Lophodermium seditiosum Epidemics in Estonia. Acta Silv. Lign. Hung.** |
| Snow Blight | *Phacidium infestans* | Pine, Fir and spruce | EU | <https://doi.org/10.1080/02827589609382912>  <https://doi.org/10.5598/imafungus.2014.05.02.02>  https://www.tandfonline.com/doi/full/10.1657/1938-4246-45.4.455 | - |
| Brown Felt Blight | *Herpotrichia juniperi* | Pine and spruce, *F*ir | EU, NA | https://www.fs.usda.gov/Internet/FSE_DOCUMENTS/stelprdb5187259.pdf | - |
| **Rhizosphaera Needle Casts** | ***Rhizosphaera pini*** | **Spruce** | **EU, NA** | [**https://www.ffpri.affrc.go.jp/pubs/bulletin/201/documents/204-3.pdf**](https://www.ffpri.affrc.go.jp/pubs/bulletin/201/documents/204-3.pdf)  [**https://www.purduelandscapereport.org/article/needlecast-diseases-not-just-a-spruce-problem/**](https://www.purduelandscapereport.org/article/needlecast-diseases-not-just-a-spruce-problem/) | **https://www.mdpi.com/1999-4907/12/4/479/htm** |
| **-** | ***Chrysomyxa ledi*** | **Spruce** | **EU, NA** | [**https://doi.org/10.1080/02827581.2010.488657**](https://doi.org/10.1080/02827581.2010.488657)  [**https://cfs.nrcan.gc.ca/publications?id=19055**](https://cfs.nrcan.gc.ca/publications?id=19055)  **doi:**[**10.1002/ece3.2056**](https://dx.doi.org/10.1002%2Fece3.2056) | **https://onlinelibrary.wiley.com/doi/10.1111/efp.12365** |
| **Powdery Mildews** | ***Microsphaera alphitoides*** | **Oak, Spruce** | **EU** | **https://www.ncbi.nlm.nih.gov/pmc/articles/PMC4866782/** [**https://www.ncbi.nlm.nih.gov/pmc/articles/PMC4410320/**](https://www.ncbi.nlm.nih.gov/pmc/articles/PMC4410320/) | **https://doi.org/10.1007/s13595-012-0252-x** |
| Venturia Leaf and Shoot Blight | *Venturia tremulae* | Poplar | EU | <https://www.fs.usda.gov/Internet/FSE_DOCUMENTS/stelprdb5191787.pdf>  https://pnwhandbooks.org/node/3308/print | - |
| Leaf Spot of Poplar | *Marssonina populi* | Poplar | EU | <https://www.jstor.org/stable/25555379>  https://www.funga.fi/Karstenia/Karstenia%2028-2%201988-3.pdf | - |
| **Septoria Canker** | **Sphaerulina musiva** | **Poplar** | **NA** | [**https://www.ncbi.nlm.nih.gov/pmc/articles/PMC4114783/**](https://www.ncbi.nlm.nih.gov/pmc/articles/PMC4114783/)  [**https://doi.org/10.2903/j.efsa.2018.5247**](https://doi.org/10.2903/j.efsa.2018.5247) | **https://doi.org/10.1094/PDIS-03-20-0494-PDN** |
| Dig deeper | Pestalotiopsis funerea | Pine and Spruce | EU | <https://doi.org/10.1111/j.1439-0329.2008.00545.x> | - |
| **European tar spots** | **Rhytisma acerinum** | **Acer** | **EU** | **doi:**[**10.1002/ece3.2056**](https://dx.doi.org/10.1002%2Fece3.2056) | **https://apsjournals.apsnet.org/doi/10.1094/PDIS-05-18-0816-PDN** |
| **Oak powdery mildew** | **Erysiphe alphitoides** | **Oak** | **Germany,**  **Poland** | **doi:**[**10.1002/ece3.2056**](https://dx.doi.org/10.1002%2Fece3.2056) | **https://doi.org/10.1007/s13595-012-0252-x** |
| Rhizina root disease | *Rhizina undulata* | Pine | EU, NA | <https://www.journals.uchicago.edu/doi/10.1086/332025>  [https://doi.org/10.1641/0006-3568(2001)051[0134:WMOEFF]2.0.CO;2](https://doi.org/10.1641/0006-3568(2001)051%5B0134:WMOEFF%5D2.0.CO;2) | - |
| **Needle cast** | ***Cyclaneusma minus*** | **Pine** | **EU NA** | [**https://www.cabi.org/isc/datasheet/35675**](https://www.cabi.org/isc/datasheet/35675) | **http://www.sipav.org/main/jpp/index.php/jpp/article/view/2382** |
| Anthracnose of plane | Apiognomonia veneta | American sycamore | EU, USA, NZ | <https://doi.org/10.1016/j.mycres.2007.03.013> | - |
| **Beech tar crust** | ***Biscogniauxia nummularia*** | **Beech** | **EU** | **Biscogniauxia nummularia infecting beech (Fagus sylvatica) trees and sympatric plants of the sedge Carex brevicollis** | **https://www.mdpi.com/1999-4907/13/1/89** |
| **Apple ring rot** | ***Botryosphaeria dothidea*** | **General** | **EU, NA** | **Urban Zdenek, 1959: Ein kleiner Beitrag zu den Rumanischen Volksrepublik, in Omagiu lui Traian Savulescu, Ed. Acad. RPR, Bucuresti, 801-810.** | **https://bsppjournals.onlinelibrary.wiley.com/doi/10.1111/mpp.12495** |
| **Bot canker of oak** | ***Botryosphaeria stevensii Shoemaker*** | **Oak and others** | **EU, NA** | **Kehr and Wulf, 1993. Fungi associated with above-ground portions of declining oaks (Quercus robur) in Germany. Eur. J, For. Path. 23: 18-27** | **https://www.nw-fva.de/fileadmin/nwfva/publikationen/pdf/langer_2021_fungi_associated_with.pdf** |
| - | *Chalara populi* | Poplar | EU | Szabó I., Harrington T.,2004. First report of Thielaviopsis populi on hybrid poplar in Hungary. Plant Pathology 53:249. | - |
| **Delphinella shoot blight** | ***Delphinella abietis*** | **Firs, Spruce** | **EU** | **Jørstad I., 1925 The Erysiphaceae of Norway, Oslo, J. Dybwad; Robak, H. (1976): Attack by Acanthostigma parasiticum (Hart.) Sacc. And Rehmiellopsis abietis (Rostr.) O. Rostrup in a provenance experiment with Abies alba. Medd. Nor. Inst. Skogf. 32(4): 167-183.** | **https://doi.org/10.3390/f7010007** |
| Stem canker of black locust | *Diaporthe oncostoma* | General | EU | Moesz, G., 1942: Pilze von Budapest und Umgebung. Kir. Magyar Természettud. Társ., Budapest. (in Hungarian) | - |
| **Cedar leaf blight** | ***Didymascella thujina*** | **Cedar** | **NA, EU** | **Pelhate J & Barbotin F 1959. Le dépérissement des thuyas et la maladie des taches foliaires dans l'Ouest, Didymascella tujina. Phytoma 104, 22-24, Lanier L 1964 Une maladie des jeunes plants de Thujas en pépiniéres. Phytoma 155: 23-27. (in lanier)** | **https://cdnsciencepub.com/doi/abs/10.1139/X07-034** |
| **Sphaeropsis blight** | ***Diplodia pinea*** | **Pine** | **EU** | **https://doi.org/10.3389/fpls.2018.01818** | **https://doi.org/10.3389/fpls.2018.01818** |
| Hypoxylon canker | *Entoleuca mammata* | Aspen, Poplar, | EU | https://link.springer.com/article/10.1007/s10342-019-01165-7 | - |
| Eutypella canker | *Eutypella parasitica* | Maple | EU | JURC, D., OGRIS, N., Slippers, B., Stenlid, J., 2006. First report of Eutypella canker of Acer pseudoplatanus in Europe. Plant Pathol., 55, 4: 577.  https://onlinelibrary.wiley.com/doi/10.1111/j.1365-2338.2006.01047.x | - |
| European Pear Rust | *Gymnosporangium sabinae* | Conifers | EU, USA | Axelsson, U. 1991, Skadegörare på plantskoleväxter, Trädgårdsrådgivningen informerar, 41  https://pubmed.ncbi.nlm.nih.gov/30764347/ | - |
| **Canker and white rot of the wood** | ***Inonotus rickii*** | **General** | **EU** | **Intini, M., (1998). Contributo alla conoscenza dei funghi lignicoli italiani: Inonotus rickii (Pat.) Reid. Micol. Ital. 20, 49-53**  [**https://www.frontiersin.org/articles/10.3389/fmicb.2021.647920/full**](https://www.frontiersin.org/articles/10.3389/fmicb.2021.647920/full) | **https://onlinelibrary.wiley.com/doi/10.1111/efp.12088** |
| Juniper Tip Blights | *Kabatina juniperi* | Pine | USA | Pettersson M-L., Åkesson I. (1998) Växtskydd i Trädgård, Natur och Kultur/LTs Förlag, pp 82, 230, 273-274  http://plantclinic.cornell.edu/factsheets/junipertipblight.pdf | - |
| European larch canker | *Lachnellula willkommii* | Pine and Spruce | EU, NA | https://www.cabi.org/isc/abstract/20056400450 | - |
| Cypress canker | *Lepteutypa cupressi* | Conifers | EU, NA | Xenopoulos G.S., 1987 A pathogen new in Greece causing the cypress canker disease, Dasiki Erevna, 8: 85-94  https://www.annualreviews.org/doi/pdf/10.1146/annurev.phyto.36.1.91 | - |
| **-** | ***Neofabraea populi*** | **Aspen** | **EU** | **Kasanen R., Hantula J., Kurkela T., 2002. Neofabraea populi in Hybrid Aspen Stands in Southern Finland Scandinavian Journal of Forest Research 17: 391-397** | **https://apsjournals.apsnet.org/doi/10.1094/PDIS-09-17-1363-RE** |
| Passalora needle blight | *Passalora sequoiae* | Conifers, cypress | NA | https://bmcresnotes.biomedcentral.com/articles/10.1186/s13104-020-05328-3 | - |
| - | *Phacidium coniferarum* | Conifers | EU | Zúbrik, M., Kunca, A., Novotný, J., 2008: Hmyz a huby : atlas poškodení lesných drevín. Národné lesnícke centrum – Lesnícky výskumný ústav Zvolen, Zvolen, 178 pp. | - |
| - | *Pithya cupressi* | Pines | EU | https://www.fs.fed.us/psw/publications/documents/psw_rp014/psw_rp014.pdf | - |
| **Shoot dieback** | ***Ramichloridium pini*** | **Pine** | **EU** | **M. A. Rahman, C. S. Millar, J. N. Gibbs (1994) Shoot dieback of lodgepole pine in Scotland caused by Ramichloridium pini. Forest Pathology 24, 40–47.** | **https://www.forestresearch.gov.uk/documents/6727/FCIN068.pdf** |
| **Cypress canker** | ***Seiridium cardinale*** | **Conifer** | **EU, NA** | **DOI: 10.1094/PDIS-12-13-1237-RE** | **DOI: 10.1094/PDIS-12-13-1237-RE**  **https://pubmed.ncbi.nlm.nih.gov/30708785/** |
| - | *Septotis podophyllina* | Poplar | EU | https://sherwoods-forests.com/Images/trees/Populus/Diseases-Of-Populus-In-BC.pdf | - |
| **-** | ***Phytophthora pluvialis*** | **Pine and Duglas-fir** | **USA and UK** | **https://www.gov.uk/guidance/phytophthora-pluvialis** | **https://www.gov.uk/guidance/phytophthora-pluvialis** |
| Sweet fern blister rust | *Cronartium comptoniae* | Pine | NA | <https://www.cabdirect.org/cabdirect/abstract/19616602315>  https://efsa.onlinelibrary.wiley.com/doi/epdf/10.2903/j.efsa.2018.5511 | - |
| - | *Pestalotiopsis pini* | Stone Pine | Italy | https://doi.org/10.3390/f11080805 | - |
| **Leaf blotch** | ***Pseudodidymella fagi*** | **European beech** | **EU** | [**https://doi.org/10.3390/f10090718**](https://doi.org/10.3390/f10090718) | [**https://doi.org/10.3390/f10090718**](https://doi.org/10.3390/f10090718) |
| Brown spot needle blight | *Mycosphaerella dearnessii* | Pine | Asia, EU, NA | <https://www.tandfonline.com/doi/abs/10.1080/07060661.2008.10540534>  https://botanicalithuanica.gamtc.lt/administravimas/uploads/b_l2011(17)129-37_602e5495dcead.pdf | - |
| - | *Naemacyclus niveus* | Pine | EU | https://doi.org/10.1111/j.1439-0329.1983.tb00119.x | - |
| - | *Cryphonectria radicalis* | Conifer | EU | https://pubmed.ncbi.nlm.nih.gov/21156482/ | - |
| - | *Collybia fusipes* | Oak | EU | Structure of Collybia fusipes populations in two infected oak stands | - |
| Brunchorstia disease | *Gremmeniella abietina* | Conifers | Eu | https://bfw.ac.at/400/pdf/fsaktuell_55_8.pdf | - |

**Table S1:** The records on forest insect pests that have and had caused outbreaks in Oak, Conifer, Birch, Poplar, Ash, Beech, Maple, Elm, and Acer trees using the keywords “forest pathogens” in Google Scholar search. The common name of the pathogens, scientific name, host trees, geographical distribution, and references are summarized. Species that have caused outbreaks are represented with the BOLD letter.

| **Common name** | **Scientific name** | **Host tree** | **Geographical range** | **References** | **Outbreak** |
| --- | --- | --- | --- | --- | --- |
| **Beech bark disease** | **Cryptococcus fagisuga** | **American beech** | **EU, NA** | [**https://esajournals.onlinelibrary.wiley.com/doi/10.1890/15-1176**](https://esajournals.onlinelibrary.wiley.com/doi/10.1890/15-1176)  [**https://www.annualreviews.org/doi/10.1146/annurev.py.32.090194.000451**](https://www.annualreviews.org/doi/10.1146/annurev.py.32.090194.000451)  [**https://www.sciencedirect.com/science/article/abs/pii/S1049964406001058?via%3Dihub**](https://www.sciencedirect.com/science/article/abs/pii/S1049964406001058?via%3Dihub) | **https://www.apsnet.org/publications/plantdisease/backissues/Documents/1985Abstracts/PD_69_905g.htm** |
| **European gypsy moth** | ***Lymantria dispar dispar* L.** | **Oaks, Aspen, Birch** | **EU, NA** | [**https://doi.org/10.1002/2688-8319.12045**](https://doi.org/10.1002/2688-8319.12045) | [**https://doi.org/10.1002/2688-8319.12045**](https://doi.org/10.1002/2688-8319.12045) |
| **Hemlock woolly adelgid** | ***Adelges tsugae* Annand** | **Hemlock** | **Asia, NA** | [**https://entnemdept.ufl.edu/creatures/TREES/hemlock_woolly_adelgid.html**](https://entnemdept.ufl.edu/creatures/TREES/hemlock_woolly_adelgid.html) | **https://mylandplan.org/content/when-you-have-hemlock-woolly-adelgid-outbreak** |
| Redbay ambrosia beetle | *Xyleborus glabratus* | redbay, sassafras, and avocado | Asia, US | <https://academic.oup.com/jee/article/101/4/1276/2198983?login=true>  https://doi.org/10.1653/024.096.0357 | - |
| **Emerald ash borer** | ***Agrilus planipennis*** | **Ash** | **EU, NA** | [**http://www.emeraldashborer.info/documents/eab_id_guide.pdf**](http://www.emeraldashborer.info/documents/eab_id_guide.pdf) | **https://www.mdpi.com/2075-4450/13/2/191** |
| **Asian longhorn beetle** | ***Anoplophora glabripennis*** | **Maple, Poplar,** | **USA, NA, EU** | [**https://www.annualreviews.org/doi/10.1146/annurev-ento-112408-085427**](https://www.annualreviews.org/doi/10.1146/annurev-ento-112408-085427)  [**https://doi.org/10.1038/s41598-019-55698-3**](https://doi.org/10.1038/s41598-019-55698-3) | **https://pflanzengesundheit.julius-kuehn.de/dokumente/upload/Anoplophora-glabripennis_pr2021-03-17_byZiemetshausen.pdf** |
| **Winter moth** | ***Operophtera brumata*** | **Oaks, and Maples** | EU | [**https://www.annualreviews.org/doi/10.1146/annurev.en.40.010195.002355**](https://www.annualreviews.org/doi/10.1146/annurev.en.40.010195.002355) | **DOI: 10.1111/j.1365-2656.2007.01339.x** |
| **Polyphagous shot-hole borer** | ***Euwallacea fornicatus*** | **More than 400 host** | **USA, SA** | [**https://link.springer.com/article/10.1007/s13313-018-0545-0**](https://link.springer.com/article/10.1007/s13313-018-0545-0) | **https://pflanzengesundheit.julius-kuehn.de/dokumente/upload/Euwallacea_fornicatus_pr_2021-12-17_NI.pdf** |
| Woodwasp | *Sirex noctilio* | Pine | EU, NA | https://doi.org/10.1017/S0007485300006155 [**https://doi.org/10.1111/j.1461-9563.2007.00340.x**](https://doi.org/10.1111/j.1461-9563.2007.00340.x) | - |
| **Southern pine beetle** | **Dendroctonus frontalis** | **Pine** | **USA, NA** | **https://doi.org/10.3390/f11020173** | **https://doi.org/10.3390/f11020173** |
| **Western pine beetle** | **Dendroctonus brevicomis** | **Pine** | **EU, NA** | **https://www.ncbi.nlm.nih.gov/pmc/articles/PMC5597856/** | **https://www.ncbi.nlm.nih.gov/pmc/articles/PMC5597856/** |
| **Eastern Spruce budworm** | **Choristoneura fumiferana** | **Spruce, Pine, Fir** | **NA** | **https://www.frontiersin.org/articles/10.3389/ffgc.2020.00014/full** | **https://www.frontiersin.org/articles/10.3389/ffgc.2020.00014/full** |
| Twig girdler | *Oncideres cingulata* | Oak, poplar  elm, birch | NA | Twig morphology and host effects on the reproductive success of the twig girdler Oncideres cingulate  https://www.fs.usda.gov/treesearch/pubs/54548 | - |
| **Eucalyptus long-horned borer** | ***Phoracantha semipunctata*** | **Eucalyptus** | **Australia,**  **Africa** | **https://www.mdpi.com/1999-4907/6/11/3868** | **https://www.mdpi.com/1999-4907/6/11/3868** |
| **Mountain pine beetle** | ***Dendroctonus ponderosae*** | **Pine** | **NA** | **https://doi.org/10.1371/journal.pone.0124691** | **https://doi.org/10.1371/journal.pone.0124691** |
| **Red turpentine beetle** | ***Dendroctonus valens*** | **Pine** | **NA, Asia** | **https://doi.org/10.1371/journal.pone.0078126** | **https://doi.org/10.1371/journal.pone.0078126** |
| **Eucalyptus snout beetle** | ***Gonipterus scutellatus*** | **Eucalyptus** | **EU** | **https://doi.org/10.2903/j.efsa.2018.5107** | **https://doi.org/10.2903/j.efsa.2018.5107** |
| - | *Gonipterus platensis* | Eucalyptus | EU | https://doi.org/10.1016/j.foreco.2022.120104 | - |
| Six-toothed bark beetle | *Ips sexdentatus* | Pine, Spruce | Eurasia | <https://www.bio-conferences.org/articles/bioconf/full_html/2019/05/bioconf_rpgrs2019_00019/bioconf_rpgrs2019_00019.html> | - |
| **Spruce bark beetle** | ***Ips typographus*** | **Pine, Spruce** | **Eurasia** | [**https://doi.org/10.1016/j.foreco.2021.119530**](https://doi.org/10.1016/j.foreco.2021.119530) | **https://doi.org/10.1111/gcb.14766** |
| **Ambrosia beetle** | ***Megaplatypus mutatus*** | **Poplar** | **SA** | [**https://doi.org/10.1093/forestry/cpm029**](https://doi.org/10.1093/forestry/cpm029) | **https://secure.fera.defra.gov.uk/phiw/riskRegister/downloadExternalPra.cfm?id=3834** |
| **Mediterranean pine beetle** | ***Orthotomicus erosus*** | **Pine** | **NA, EU** | **https://www.seefor.eu/vol-10-no-1-pernek-et-al-outbreak-of-orthotomicus-erosus.html** | **https://www.seefor.eu/vol-10-no-1-pernek-et-al-outbreak-of-orthotomicus-erosus.html** |
| Oak pinhole borer | *Platypus cylindrus* | Oak | EU, Asia | https://journalissues.org/ijapr/wp-content/uploads/sites/5/2021/01/Amoura-et-al.pdf | - |
| Pine shoot beetle | *Tomicus piniperda* | Pine | EU, NA | Occurrence, performance and shoot damage of *Tomicus piniperda* in pine stands in southern Sweden after storm-felling  https://www.sciencedirect.com/science/article/pii/S0378112711004737 | - |
| **Cockchafers** | ***Melolontha* spp** | **Oak, Pine** | **EU** | **https://link.springer.com/article/10.1007/s10526-019-09927-3** | **https://link.springer.com/article/10.1007/s10526-019-09927-3** |
| - | *Cinara cupressivora* | Pine | NA | Occurrence of *Cinara* spp. (Hemiptera, Aphididae) on *Pinus* spp. (Pinaceae), in the county of Lages-SC, Brazil. | - |
| - | *Cinara pinivora* | Pine | NA | https://pdfs.semanticscholar.org/addf/92a5da24061312ff2dbbdf934abf68981a26.pdf | - |
| **-** | ***Heteropsylla cubana*** | **General** | **NA** | **Studies on the outbreak of leucaena psyllid (Heteropsylla cubana Crawford) on *Leucaena leucocephala* (Lam.) de Wit cv. Tarramba in Thailand [2011]** | **Studies on the outbreak of leucaena psyllid (Heteropsylla cubana Crawford) on *Leucaena leucocephala* (Lam.) de Wit cv. Tarramba in Thailand** |
| Oak lace bug | *Corythucha arcuata* | Oak | NA, EU | https://pureportal.spbu.ru/en/publications/first-documented-outbreak-and-new-data-on-the-distribution-of-cor | - |
| Eucalyptus gall wasp | *Leptocybe invasa* | Eucalyptus | NA | An overview on outbreak of Eucalyptus gall wasp, Leptocybe invasa (Hymenoptera: Eulophidae) in Northern India. | - |
| **Eucalyptus gall wasp** | ***Ophelimus maskelli*** | **Eucalyptus** | **Asia,**  **NA** | **CSL PEST RISK ANALYSIS FOR OPHELIMUS MASKELLI** | **CSL PEST RISK ANALYSIS FOR OPHELIMUS MASKELLI** |
| **Fall cankerworm** | ***Alsophila pometaria*** | **Oak** | **NA** | **https://doi.org/10.1093/ae/tmv043** | **https://doi.org/10.1093/ae/tmv043** |
| Siberian moth | *Dendrolimus sibiricus* | Spruce, Pine | Eurasia | https://forestecosyst.springeropen.com/articles/10.1186/s40663-020-00258-9 | - |
| **Tent caterpillar** | ***Malacosoma disstria*** | **Poplar** | **NA** | **https://doi.org/10.3389/fevo.2015.00078** | **https://doi.org/10.3389/fevo.2015.00078** |
| **White satin moth** | ***Leucoma salicis*** | **Poplar and willow** | **EU** | **https://doi.org/10.2478/v10045-008-0004-y** | **https://doi.org/10.2478/v10045-008-0004-y** |
| **Nun moth** | ***Lymantria monacha*** | **Conifer** | **EU, NA** | **Outbreak of nun moth (Lymantria monacha L.) in Poland between 1978 and 1984 [1986]** | **Outbreak of nun moth (Lymantria monacha L.) in Poland between 1978 and 1984 [1986]** |
| Mahogany shoot borer | *Hypsipyla grandella* | Cedar | South America | Antifeedant activity of *Quassia amara*(Simaroubaceae) extracts on *Hypsipyla grandella* (Lepidoptera: Pyralidae) larvae | - |
| **Western spruce budworm** | ***Choristoneura occidentalis*** | **Pine, balsam fir and**  **spruce** | **NA** | **https://www.fs.usda.gov/treesearch/pubs/6390** | **https://www.fs.usda.gov/treesearch/pubs/6390** |
| Jack-pine budworm | *Choristoneura pinus* | Pine, and  spruce | North America | https://link.springer.com/article/10.1007/s13355-012-0097-7 | - |
| **Oak-leaf roller** | ***Tortrix viridana*** | **Oak** | EU | [**https://eurekamag.com/research/027/487/027487534.php**](https://eurekamag.com/research/027/487/027487534.php) | **https://doi.org/10.1111/j.1439-0418.1984.tb02687.x** |
| **Pine processionary** | ***Thaumetopoea bonjeani*** | **Cedar** | **Africa** | **https://doi.org/10.1080/09583157.2016.1160029** | **https://doi.org/10.1080/09583157.2016.1160029** |
| **-** | ***Thaumetopoea pityocampa*** | **Pine and cedar** | **EU** | **The outbreak of the pine processionary moth in Venosta/Vinschgau: ecological and economic aspects** | **The outbreak of the pine processionary moth in Venosta/Vinschgau: ecological and economic aspects** |
| **Oak processionary moth** | ***Thaumetopoea processionea*** | **Oak, Pine** | **EU** | **An Epidemic Airborne Disease Caused by the Oak Processionary Caterpillar**  **https://doi.org/10.1111/j.1461-9563.2011.00552.x** | **An Epidemic Airborne Disease Caused by the Oak Processionary Caterpillar**  **https://doi.org/10.1111/j.1461-9563.2011.00552.x** |
| **Beech scale** | **Cryptoccocus fagisuga** | **Beech** | **EU** | **https://doi.org/10.1603/0046-225X-33.5.1274** | **https://doi.org/10.1603/0046-225X-33.5.1274** |
| Balsam Woolly Adelgid | *Adelges piceae* | Fir | EU | Kotinsky, Jacob. "The European fir trunk bark louse (Chermes (Dreyfusia) piceae Ratz.) apparently long established in the United States." Proceedings Entomological Society of Washington 18 (1916): 14-16. | - |
| Red Pine Scale | *Matsucoccus matsumurae* | Pine | NA, Asia | Bean, James L., and Paul A. Godwin. "Description and bionomics of a new red pine scale, Matsucoccus resinosae." Forest Science 1.2 (1955): 164-176. | - |
| European Pine Needle Midge | Contarinia baeri | Pine | EU | Adams, LE 1975. A new midge, Contarinia baeri (Prell), on Scotch pine in Pennsylvania. Pa. Christmas Tree Bull. No. 127 (Mar.), p. 5. |  |
| **Red-haired Pine Bark Beetle** | ***Hylurgus ligniperda*** | **Pine** | **US, EU** | **Hoebeke, E. Richard. "Hylurgus ligniperda: a new exotic pine bark beetle in the United States." Newsl. Mich. Entomol. Soc 46.1-2 (2001): 1-2.** | **https://www.scielo.cl/scielo.php?script=sci_arttext&pid=S0717-92002017000100006&lng=en&tlng=en** |
| **Basswood Thrips** | ***Thrips calcaratus*** | **Conifers** | **US, EU** | **https://doi.org/10.1016/j.foreco.2005.12.052** | **https://doi.org/10.1016/j.foreco.2005.12.052** |
| European Bark Beetle | *Hylastes opacus* | Conifers | US, EU | Wood, S.L. 1992. Nomenclatural changes and new species in , Platypodidae and Scolytidae Coleoptera), Part 11. Great Basin Nat. 52: 78-88. | - |
| Willow Leaf Beetle | *Plagiodera versicolora* | Willow | US, EU | Schaeffer, Charles. "New Coleoptera and miscellaneous notes. III." Journal of the New York Entomological Society 23.4 (1915): 235-238. | - |
| **Five-spiked bark beetle** | ***Ips grandicollis*** | **Pine** | **Australia** | **https://doi.org/10.1111/aen.12077** | **https://doi.org/10.1111/aen.12077** |
| Elongate Hemlock Scale | *Fiorinia externa* | Conifers | US, Asia | Sasscer, Ernest Ralph. The genus Fiorinia in the United States. No. 16. US Government Printing Office, 1912. | - |
| Japanese Cedar Longhorn Beetle | *Callidellum rufipenne* | Conifers | USA, Asia | Hoebeke, E. Richard. "Japanese cedar longhorned beetle in the eastern United States." (1999). USDA Pest Alert. APHIS 81-35-004 | - |
| Pine bark anobiid | *Ernobius mollis* | Pine, Spruce | EU | Milligan, R.H. 1977: *Ernobius mollis* Linnaeus (Coleoptera: Anobiidae), pine bark anobiid. *New Zealand Forest Service, Forest and Timber Insects in New Zealand No.17.* | - |
| Queensland pine beetle | *Calymmaderus incisus* | Pine | Australia | https://www.business.qld.gov.au/industries/farms-fishing-forestry/forests-wood/pests-diseases/trees-timber/queensland-pine-beetle | - |
| Oak Wilt | *Ceratocystis fagacearum* | Oak | UK, USA | <https://www.cabi.org/isc/datasheet/12142>  https://www.forestresearch.gov.uk/documents/7318/Contingency_plan_-_oak_wilt_FINAL_30-01-17.pdf | - |
| Neonectria canker of fir | *Neonectria neomacrospora* | Fir | EU NA | https://pflanzengesundheit.julius-kuehn.de/dokumente/upload/Neonectria-neomacrospora_pr2018BB.pdf | - |
| Two-spotted oak buprestid | *Agrilus biguttatus* | Oak | EU | https://www.canr.msu.edu/ipm/uploads/files/forecasting_invasion_risks/oaksplendorbeetle.pdf | - |
| **Pine-tree lappet moth** | ***Dendrolimus pini*** | **Pine** | **EU NA** | **https://doi.org/10.1016/j.foreco.2019.117697** | **https://doi.org/10.1016/j.foreco.2019.117697** |
| **Large pine weevil** | ***Hylobius abietis*** | **Pine** | **EU** | **https://doi.org/10.1017/S0007485399000024** | **https://doi.org/10.1017/S0007485399000024** |
| **Horse-chestnut leaf miner,** | ***Cameraria ohridella*** | **Acer, Maple** | **EU, UK** | [**https://doi.org/10.1111/j.1439-0418.2005.00973.x**](https://doi.org/10.1111/j.1439-0418.2005.00973.x) | **https://www.researchsquare.com/article/rs-466202/v1** |
| Great spruce bark beetle | *Dendroctonus micans* | Pine and Spruce | EU, UK | https://doi.org/10.1016/j.foreco.2004.05.059 | - |
| Elm zigzag sawfly | *Aproceros leucopoda* | Elm | UK | https://jhr.pensoft.net/articles.php?id=4395 | - |
| **Citrus longhorn beetle** | ***Anoplophora chinensis*** | **Poplar and Oak** | **EU** | **https://www.semanticscholar.org/paper/ANOPLOPHORA-CHINENSIS-(FORSTER)-(COLEOPTERA-IN-THE-Peverieri-Bertini/3eaae732a5f4cb59a9e40d0562e2826011811a36** | **https://www.semanticscholar.org/paper/ANOPLOPHORA-CHINENSIS-(FORSTER)-(COLEOPTERA-IN-THE-Peverieri-Bertini/3eaae732a5f4cb59a9e40d0562e2826011811a36** |
| Bronze birch borer | *Agrilus anxius* | Birch | NA | https://pflanzengesundheit.julius-kuehn.de/dokumente/upload/agrlax-steckbrief.pdf | - |
| **Red Oak Borer,** | ***Enaphalodes rufulus*** | **Oak** | **NA** | [**http://hyg.ipm.illinois.edu/article.php?id=648**](http://hyg.ipm.illinois.edu/article.php?id=648) | **https://doi.org/10.1139/X10-028** |
| laminated root rot | *Phellinus weirii* | Douglas-fir | NA | https://www.cabi.org/isc/datasheet/28733#todistributionDatabaseTable | - |
| ***Chinese White Pine Beetle*** | ***Dendroctonus armandi*** | **Pine** | **Asia** | **https://doi.org/10.3389/fphys.2020.546592** | **https://doi.org/10.3389/fphys.2020.546592** |
